# Thin Degradable Coatings for Optimization of Osteointegration Associated with Simultaneous Infection Prophylaxis

**DOI:** 10.3390/ma12213495

**Published:** 2019-10-25

**Authors:** Sophie Burtscher, Peter Krieg, Andreas Killinger, Ali Al-Ahmad, Michael Seidenstücker, Sergio Hernandez Latorre, Anke Bernstein

**Affiliations:** 1 G.E.R.N. Tissue Replacement, Regeneration & Neogenesis, Department of Orthopedics and Trauma Surgery, Medical Center, Faculty of Medicine, Albert-Ludwigs-University of Freiburg, Hugstetter Straße 55, 79106 Freiburg, Germany; sophie.burtscher@posteo.net (S.B.); michael.seidenstuecker@uniklinik-freiburg.de (M.S.); sergio.latorre@uniklinik-freiburg.de (S.H.L.); 2 Institute for Manufacturing Technologies of Ceramic Components and Composites (IMTCCC), University of Stuttgart, Allmandring 7b, 70569 Stuttgart, Germany; peter.krieg@ifkb.uni-stuttgart.de (P.K.); andreas.killinger@ifkb.uni-stuttgart.de (A.K.); 3 Department of Operative Dentistry and Periodontology, Center for Dental Medicine, Faculty of Medicine, Albert-Ludwigs-University, Hugstetter Straße 55, 79106 Freiburg, Germany; ali.al-ahmad@uniklinik-freiburg.de

**Keywords:** calcium phosphate, bioglass, antimicrobial active metals, coating, high velocity flame suspension spraying, biocompatibility

## Abstract

One of the most common causes of implant failure is aseptic prosthesis loosening. Another frequent complication after prosthesis implant is the microbial colonization of the prosthesis surface, which often leads to a replacement of the prosthesis. One approach to reduce these complications is the application of bioactive substances to implant surfaces. Both an antibiotic prophylaxis and a faster osteointegration can be obtained by incorporation of bactericidal active metals in degradable calcium phosphate (CaP) coatings. In this study, thin degradable calcium phosphate ceramic coatings doped with silver (Ag), copper (Cu), and bismuth (Bi) on a titanium substrate were prepared with the aid of the high-velocity suspension flame spraying (HVSFS) coating process. To characterize the samples surface roughness, brightfield microscopy of the coatings, X-ray diffraction (XRD)-analysis for definition of the phase composition of the layers, Raman spectroscopy for determination of the phase composition of the contained metals, element-mapping for Cu-content verification, release kinetics for detection of metal ions and ceramic components of the coatings were carried out. The aim of this study was to evaluate in vitro biocompatibility and antimicrobial activity of the coatings. For biocompatibility testing, growth experiments were performed using the cell culture line MG-63. Cell viability was investigated by Giemsa staining and live/dead assay. The WST-1 kit was used to quantify cell proliferation and vitality in vitro and the lactate dehydrogenase (LDH) kit to quantify cytotoxicity. The formation of hydroxyapatite crystals in simulated body fluid was investigated to predict bioactivity in vivo. The Safe Airborne Antibacterial Assay with *Staphylococcus aureus* (*S. aureus*) was used for antimicrobial testing. The results showed good biocompatibility of all the metal doped CaP coatings, furthermore Cu and Ag doped layers showed significant antibacterial effects against *S. aureus*.

## 1. Introduction

Arthroplasty is an effective treatment for severe trauma and arthritic joint diseases. Aseptic loosening has been acknowledged as one of the leading causes of revisions procedures in patients with previous joint arthroplasty [1]. This phenomenon is attributable to the missing induction of secondary stability; meaning that the firm anchorage needed between the prosthesis and the surrounding bone on a long term is missing [2,3]. Another common complication after implantation is microbial colonization on the prosthesis surface [4]. These complications often lead to secondary surgery and implant-replacement being required [1]. In order to reduce these complications, implant surfaces can be coated with bioactive substances which can have both osteoconductive and antimicrobial properties [5]. In this study 12 different biodegradable coatings were characterized and examined with regard to their biocompatibility and antimicrobial efficacy. Each coating consists of a resorbable osteoconductive ceramic component, and an antimicrobial-active metal component. Hydroxyapatite (HA), β-tricalcium phosphate (TCP), a glass ceramic (GB14) and bioglass were used as ceramic components. Those ceramics have in common to have a high CaO/P_2_O_5_ content. They therefore correspond to the inorganic parts of mineralized bone, show good biocompatibility and osteoconductivity and are commonly used for coating joint implants [6,7,8]. Clinical experience in recent years has shown that biomaterials containing important bone minerals enhance stable anchorage of the implant in the surrounding bone. Direct bonding of bone to the ceramic surface is the most important precondition for durable and stable osseointegration. Their speed of degradation should ideally be synchronized with bone regeneration to ensure bone maturation and sufficient stability at the interface. In addition, these materials must have the ability to shorten the time between primary stability (the strength of fixation between bone and implant achieved during surgery) and secondary stability (definitive bone integration [9]).

Biodegradable materials such as CaP ceramics are also potential carriers for antimicrobial substances: through their gradual decomposition, a continuous release of antimicrobial active substances such as metal ions can be maintained over longer periods of time [10]. As antimicrobial active metal components silver (Ag), copper (Cu) and bismuth (Bi) were chosen, since their antimicrobial virtues are known and described in literature [11,12,13]. These metals showed to be appropriated to be integrated in the coatings. 

A doping of CaP coatings with foreign metal ions such as silver [14,15] or copper [14,16,17] leads to the release of these ions from the layer. These ions have antibacterial properties to counteract the colonization of the implant surface with microorganisms and thus the formation of a biofilm. Silver is relatively safe in local application, free of side effects [18], has the highest toxicity to microorganisms and has a low risk of resistance formation [19]. Silver ions kill the bacteria by influencing the membrane function [20]. Very small quantities are therefore sufficient. The effective concentrations of silver ions against bacteria, fungi and viruses range from 10^−9^ to 10^−6^ mol/L [19,21]. These low concentrations do not cause toxic effects on human cells [19,22]. Coatings with elemental silver show a good effect against *Staphylococcus aureus* in animal experiments as well as in tumor endoprosthetics [22,23]. The antimicrobial effect of copper ions and its influence on biofilm formation [24] are comparable to that of silver ions [25]. Bismuth ions also have this property [26]. They have been used for years as root canal filling materials [27]. Only recently it has been shown that bismuth compounds have antibacterial effects as additives in CaP cements [28].

A combination of the osteoconductive ceramic and the antimicrobial metal-component was applied on pure titanium plates via high velocity flame suspension spraying (HVSFS) in order to obtain thin coatings with 20 µm thickness. Layer thickness determines the mechanical properties of the coating. Layer thicknesses between 20 and 50 μm proved to be optimal for coating orthopaedic implants [29,30,31] since layers in this size range exhibit high corrosion resistance and adhesive strength [32]. With thicker layers >100 μm ceramic fractures as well as fragmentation and detachment as a result of shear-, bending-, tensile- and compressive forces occur more frequently [32,33,34]. On the other hand thin layers in the nanometer range <1 μm, increase the risk of undesired prosthesis migration within the bone (shaft migrations) and thus jeopardize the long-term stability of the prosthesis [34]. We have succeeded in establishing the necessary process for the HVSFS suspensions and in obtaining very finely structured, thin homogeneous layers. With the help of such coatings a faster ingrowth and significantly improved mechanical stability after operation is to be achieved. The materials without metal additive show no change in biocompatibility after deposition on a metal substrate. Both an antibiotic prophylaxis and a faster osteointegration can be obtained by incorperation of bactericidal active metals in degradable CaP coatings. In this study the release kinetics of the various ions and the biocompatibility in vitro, and the antibiotic effectiveness of the spray coatings were investigated.

## 2. Materials and Methods

The chemical composition, the particle size and the provider of the used ceramics are listed in Table 1. 

The copper (Cu(CH_3_COO)_2_), silver (AgNO_3_) and pure bismuth particles were provided by Alfa Aesar (Karlsruhe, Germany). The elaboration and processing of the coatings was done by the Institute for Manufacturing Technologies of Ceramic Components and Composites (IMTCCC, Stuttgart, Germany) of the University of Stuttgart. As control samples pure titanium (purity level 2, Zapp AG, Ratingen, Germany) samples with a surface roughness of Sa = 5.85 μm were carried along in each experiment. To obtain samples sized 1 × 1 cm in measure to carry out the experiments in 24 well plates, the originally 5 × 5 cm sized samples were cut using a pathology saw type Cut Grinder (Walter Messner GmbH, Oststeinbek, Germany) with a diamond saw blade type Cut Grinder D151 (Walter Messner GmbH, Oststeinbek, Germany), 3 mm thickness. During the procedure sample and sawblade were constantly cooled by gently running water. After the cutting process the samples were manually rinsed with distilled water and air dried. 

### 2.1. HVSFS Coating Process

The HVSFS process was developed from high velocity oxifuel spraying (HVOF) and is based on micrometer, submicron and nanoscale particles such as HA, TCP, GB14 and bioglass ceramic powders or metal salts are brought in suspension with a solvent and a dispersing agent. The suspension is then projected axially at supersonic speed onto a substrate after passing through a flame, which melts the powder components. The exact composition of the suspension for the coatings can be found in Table 2. For this thermal spray process lots of process parameters determine the structure of the produced coatings: droplet size, injection speed, velocity fields in the combustion chamber, solvent selection and many more [20]. A more thorough description of the HVSFS process and setup can be found elsewhere [21].

### 2.2. Sample Characterization

#### 2.2.1. Surface Roughness

To determine the surface roughness the samples were examined using KEYENCE’s VKX-210 3D laser scanning microscope (Keyence, Osaka, Japan) with Nikon objectives. Measurements were carried out in 200-, 400- and 1000-fold magnification. The measurements were evaluated using the Keyence VK analysis module (V3.5.0.0, Keyence, Osaka, Japan). The main focus was on the surface roughness (S_a_), the entire surface of a cut sample (sized 0.904 ± 0.08659 cm²) was examined. 

#### 2.2.2. Brightfield Microscopy of the Coatings 

Brightfield microscopy was done for visual detection of the metal doping in the coatings. At the same time the coating-thickness was measured. For taking the bright field microscopy shots the samples were coated with a 1 nm layer of Pt/Pd (80/20). The images were taken at 3 kV with a Scanning electron microscope (SEM) Zeiss DSM 982 Gemini Microscope (Zeiss, Oberkochen, Germany) at the IFKB Stuttgart. 

#### 2.2.3. XRD-Analysis for Definition of the Phase Composition of the Layers 

X-ray diffraction measurement (XRD) allows determination of phase composition and crystallinity of materials using X-ray diffraction phenomena. Such a measurement was performed on the coatings after the spraying process, as some spray parameters can influence the phase composition. The measurements were performed by Bruker AXS D8 Advance Diffractometer (Bruker AXS Inc., Madison, WI, USA) at 40 kV and 40 mA at IMCCCT Stuttgart. The scanning was done in 2θ from 20–80° with a scanning step of 0.01. The DiffracPlus Eva software was used for the evaluation. 

#### 2.2.4. Raman Spectroscopy for Determination of the Phase Composition of the Metals

Raman microscopy was used to determine whether the pure metals or their compounds were still present in the coatings after the spraying process. The measurements were also performed at the IMCCCT Stuttgart with a Raman microscopy spectrometer (XploRA, Horiba Instruments, Kyoto, Japan). The plates were examined microscopically and spectroscopically at selected measuring points: The spots that appeared coloured under the microscope were chosen for measurement. The measurements were carried out using a confocal measuring system in order to carry out depth measurements (AviSpectro, Stuttgart, Germany). The parameters are shown in Table 3.

#### 2.2.5. SEM for Verification Metal Contained in the Coatings 

Scanning electron microscope (SEM) images were taken by the IMCCCT (University of Stuttgart, Stuttgart, Germany) to provide qualitative evidence of the metal doping in the coatings. Therefore a high field emission gun SEM DSM 982 Gemini (Zeiss) was used (3–10 kV). Heavier elements have a different aspect than lighter elements such as calcium and phosphate.

#### 2.2.6. Element-Mapping for Cu-Content Verification 

Element mapping images are mainly used for the spatial representation and zoning of various elements contained in a sample. The copper-containing layers were examined by means of this method, since the doping can be recognized clearly within the coatings. A SEM DSM 982 Gemini high field emission gun (LEO Elektronenmikroskopie GmbH, Oberkochen, Germany) was used.

#### 2.2.7. Release Kinetics for Detection of Metal Ions and Ceramic Components of the Coatings

To determine the release of coating components into water a release kinetics experiment was undertaken. Samples were placed in labeled sealable jars (VWR, Radnor, PA, USA), the coating in direction of the opening. Six mL of ultrapure water were then pipetted into each receptacle. The jars were then placed lid closed on the shaker at 180 rpm and 37 °C room temperature. After 6 h, 6 mL were taken from each glass, put into a tube and immediately frozen. The jars were filled up again with 6 mL ultrapure water and placed on the shaker at 37 °C. After 24 h, 48 h, 72 h the same procedure was followed: freezing 6 mL from each sample and refilling the receptacle with 6 mL ultrapure water.

The Ag, Cu and bismuth ion concentrations of the samples taken were then measured by atomic absorption spectroscopy (AAS ZEEnit 650, Analytik Jena, Jena, Germany) at the Institute for Environment and Natural Sciences, Department of Geochemistry. The measurement of the calcium, phosphate, potassium and sodium content of the samples was carried out in the Department of Clinical Chemistry of the University of Freiburg. The ions were quantified by flame spectrometry in a Cobas 8000 (Roche, Hamburg, Germany) calibrated to human blood plasma. 

The total amount of metal contained in a sample before the release-experiment was determined. This value was then put into relation with the amount released during this experiment. From a sample sized 1 cm² about 0.01 g coating material could be manually scraped off with a scalpel. The suspension with which the coating was produced consisted of 10% solids, of which 1.75% was metal. That means that in 0.01 g coating approx. 9.825 mg ceramic and 0.175 mg metal/metal compound were contained. Thus the following quantities of pure metal were integrated into the coatings (Table 4).

### 2.3. Biocompatibility Testing in Vitro

#### 2.3.1. Cell Culture

For biocompatibility testing in vitro cell culture experiments were carried out using MG-63 cells (ATCC CRL-1427). The cells were maintained in a default medium constituted of Dulbeccos Modified Eagle Medium MEM/F-12 (DMEM F-12) (Lonza, Waldshut-Tiengen, Germany) supplemented with 1% penicillin/streptomycin (Gibco, Braunschweig Germany) and 10% fetal bovine serum (Biochrom, Berlin, Germany). The cell line was subcultured by trypsin/ethylene diamine tetracetic acid treatment (Gibco, Braunschweig, Germany) twice a week, the medium was replaced every second day. The cells were kept in an incubator at 37°, 5% CO2 and 100% humidity. For each experiment four sets of samples were examined and each experiment was carried out three times.

For the seeding process the samples including a titanium control were placed on labelled petri dishes. A cell-suspension of 5000 cells in 75 µL default medium was pipetted on each sample. After 2 h incubation and precipitation of the cells on the samples surface, the samples were transferred in 24-well plates and supplemented with 1 mL default medium.

#### 2.3.2. Cell Viability

Live dead assay is a method to visualize living or dead cells using fluorescent dyes. The calcein contained in the kit is lipophilic and can penetrate cell membranes of vital cells and dyes them green. On the other hand ethidium homodimer contained in the kit, can only penetrate cells whose cell membrane is damaged. It binds to the cell DNA and stains it red.

After 3, 7, 14 and 21 days of incubation, the live/dead cell staining Kit II (Roche Diagnostics GmbH, Mannheim, Germany) was used on the samples according to the manufacturer’s protocol. It allows the vital cells to be stained green using calcein AM and the dead cells red using ethidium homodimer III (EthD-III) together with an effective plasma membrane. Two mL PBS (Gibco, Braunschweig Germany) were mixed with 4 μL ethidium homodimer 12 mM in DMSO/H_2_O 1:4. One μL 4 mM calcein was put in anhydrous DMSO (Sigma-Aldrich, Steinheim, Germany). The samples were then washed three times in PBS, mixed with 50 μL of the kit mixture and incubated for 10 minutes. After the dying process the samples were examined using a fluorescence microscope (Olympus BX-51, Olympus, Hamburg, Germany) and dead and alive cells were counted out. 

#### 2.3.3. WST-1-Kit Was Used to Quantify Cell Proliferation and Viability

MG-63 was tested using the cell proliferation reagent WST-1-Kit (Roche Diagnostics GmbH, Mannheim, Germany). The conversion of tetrazolium salt via mitochondrial succinate-dehydrogenase-vital cells to colored formazan was measured spectrophotometrically. For each day three scaffolds were examined. The Assay was repeated three times.

The medium was removed from the microtiter plates after 3, 7, 14, and 21 days and the sample bodies were transferred to a new 24-well-plate. Both the original and the new well plate were then supplemented with 600 µL phenol-free standard medium (Gibco, Paisley, UK) and 60 µL WST-reagent. The plates were then incubated for 2 h at 37 °C and 5% CO_2_. Finally, 4 × 100 µL of the supernatant was pipetted out of every well into a 96 well plate. The absorption of the liquid at a wavelength of λ = 450 nm and λ = 600 nm reference was measured via an ELISA-Reader (Tecan Deutschland GmbH, Crailsheim, Germany).

#### 2.3.4. Lactate Dehydrogenase (LDH) Test to Determine Cytotoxicity

We analyzed the cytotoxicity on MG-63 using the Cytotoxicity Detection Kit (Roche Diagnostics GmbH, Mannheim, Germany). It relies on the fact that the plasma membrane of dead or damaged cells is no longer intact and that cytoplasmic enzyme lactate dehydrogenase (LDH) is thereby released. As suggested and described by the manufacturer [35] the optimal cell count for the LDH-Test was determined. An initial cell count of 50,000 cells suspended in 75 µL phenol-free medium was chosen and pipetted onto the samples. A background control provided information about the LDH activity contained in the assay medium. The absorbance value obtained in this control was subtracted from all other values. A low control (titanium with cell suspension) provided information about the LDH activity released from the untreated normal cells. A high control provided information about the maximum releasable LDH activity in the cells. For the high control cells pipetted onto a pure titanium sample were lysated by adding 10 µL Triton- X (Sigma-Aldrich, Steinheim, Germany) to the well. The seeded samples were incubated 24, 48 and 72 h at 37 °C, 5% CO_2_ and 100% humidity. After incubation 4 × 100 µL of the supernatant was taken out of every well and put into a 96-well plate and the LDH-Test performed according to the manufacturer’s instructions. Absorption of the liquid was read at a wave length of λ = 490 nm and a reference of λ = 600 nm. The obtained values were then converted into cytotoxicity values according to the formula provided by the kit manufacturer. 

#### 2.3.5. In Vivo Bone Biocompatibility via Simulated Body Fluid (SBF) Experiment

Simulated Body Fluid was made as described by Kokubo et al. [36]. Two sets of samples were placed in well plates, covered with SBF and incubated at 37 °C, 5% CO_2_ and 100% humidity. After 14 days of incubation one set of samples was rinsed with distillated water and air dried, same happened with the second set after 28 days of incubation. The samples were then visualized photo-microscopically (Olympus BX-51) and electron-microscopically (ESEM Fei Quanta 250 FEG, Fei, Hillsboro, WA, USA) to detect Hydroxyapatite crystals. XRD-Analysis (Bruker AXS D8 Advance Diffractometer) at 40 kV and 40 mA at IFKB Stuttgart was done to objectify the composition of the found crystals. 

### 2.4. Antimicrobial Testing

For examination of the antimicrobial virtues of the samples on *Stapylococcus aureus* (ATCC29593) Safe Airborne Antibacterial Assay (SAAA) was carried out. The method was applied according to the developers Al-Ahmad et al. The exact setup of the experiment is described elsewhere in detail [37]. For this experiment a suspension of 10^7^ germs in Tryphone Soya Broth (TSB) Medium (Oxoid, Altrincham, UK) was produced. One mL of the suspension was mixed with 100 mL sterile 0.9% NaCl solution (B. Braun, Melsungen, Germany) and put into the glass bulb of the Standard Chromatography Sprayer. Every sample was fixed onto a Petri dish with double sided tape, and placed in 15 cm distance of the Standard Chromatography spray head. With an air-volume of a twice filled 60 cm^3^ syringe, the samples were sprinkled with the bacterial suspension. The petri dishes with the samples were incubated 30 min at 37° 5% CO_2_ and 100% humidity. The samples were then wetted with 50 µL 0.9% NaCl solution and again incubated for 2 min. The liquid obtained on the samples surface was spread onto fresh Columbia Agar Plates with 5% sheep blood (Oxoid, Altrincham, UK). After 12 to 24 h incubation the number of bacterial colonies growing on the plates was counted out. 

### 2.5. Statistical Analysis

The results of the experiments are indicated as mean values ± standard deviation. In a first step the distribution of the values was tested. The normality test was performed graphically and by means of the Kolmogorov-Smirnov test. The Mann-Whitney U-test was used to compare two independent groups. The following variables of the examined samples were included:

- Time of data collection (3, 7, 14, 21 days or 24, 48, 72 h)
- Ceramic component (TCP, HA, GB14, bioglass, titanium)
- Metal doping (Cu, Ag, Bi, no doping)

The abovementioned testing as well as the graphical representations and the statistical significance calculation were carried out with SPSS statistics program (version 22.0, IBM, Armonk, NY, USA). Results showing a significance level of p < 0.05 results were considered to be significant. 

## 3. Results

### 3.1. Sample Characterization

**Figure 1:**
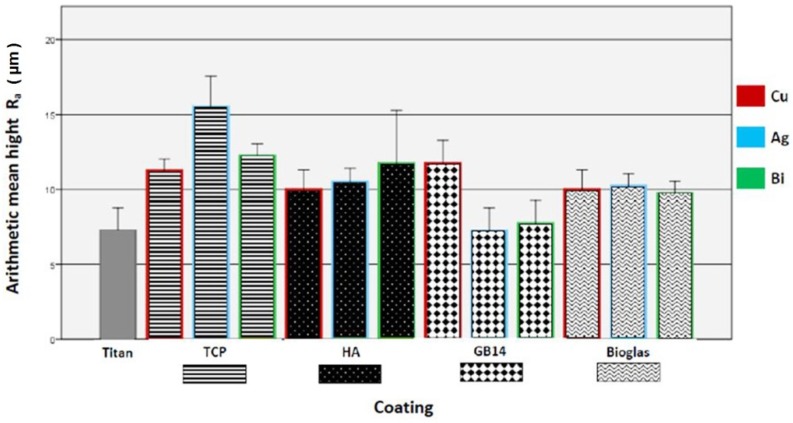
Surface roughness of the coatings, sorted by ceramic groups. Red bordered: copper-doped, blue bordered: silver-doped, green bordered: bismuth-doped.

The surface roughness values were found to be in a range from 7.25 µm (GB14 + Ag) to 15.50 µm (TCP + Ag) as can be seen in Figure 1, the exact values can be found in the supplement (Figure S1 and Table S1). The highest surface roughness was found on TCP + Ag, followed by TCP + Bi. The lowest surface roughness was found on GB14 + Ag and GB14 + Bi. 

#### 3.1.1. Brightfield Microscopy of the Coatings

**Figure 2:**
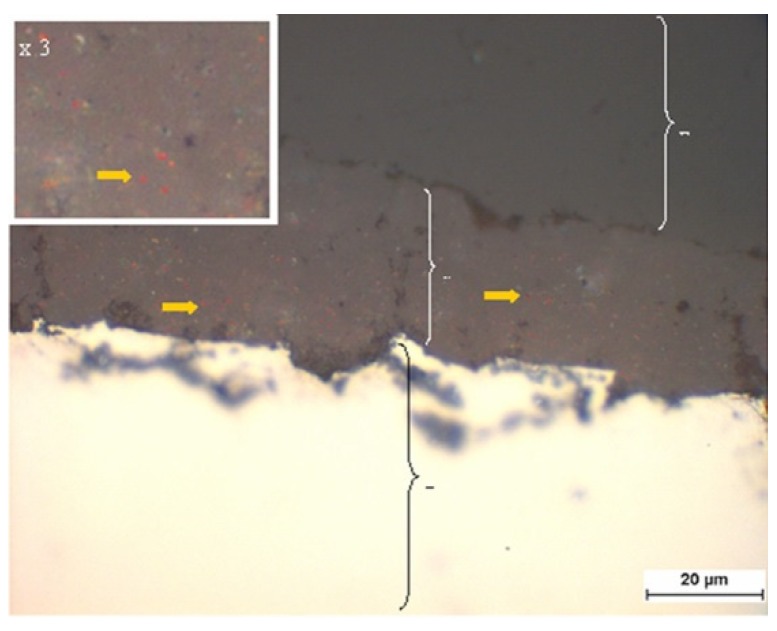
Surface morphology using high-resolution field emission gun, 1000× magnification, GB14 + Cu. C =coating, T = titanium substrate, the yellow arrows point out the reddish Cu particles. The white bar corresponds to 20 µm.

The metal particles incorporated in the layers, especially copper, are recognizable by their reddish color, as can be seen on the example of the GB14 + Cu sample (yellow arrows in Figure 2) The coatings were shown to have an uniform structure and a low porosity, as can also be seen in the example of GB14 + Cu. The layer thicknesses varied from 10.2 µm ± 1.15 (GB14 + Cu) to 29.7 ± 3.24 (GB14 + Bi) further values and images can be found in the Supplementary Material (Table S2). 

#### 3.1.2. XRD Analysis

All XRD-analysis graphs can be found in the Supplementary Material (Figures S2–S5). The XRD spectrum of HA coatings showed calcium phosphates of different stoichiometric compositions: (Ca_9.42_Sr_0.18_H_0.4_ (PO_4_)_6_ (OH)_1.6_ and Ca_9.74_ (P O_4_)_6_ (OH)_2.08_) were found as the main crystalline phases. Titanium as the substrate could also be detected through the thin coating layer. 

In TCP coatings, mainly TCP (Ca_3_(PO_4_)_2_) in α and β-form was detectable. Further stoichiometrically differently composed calcium phosphates were also detected (Ca_9.74_(PO_4_)_6_ (OH)_2.08_ and Ca_9.04_(PO_4_)_6_ (OH)_1.68_). In GB14 coatings KNaCa_2_(PO_4_)_2_ was found as the main phase. In the GB14 + Bi coating HA and Ca_3_ (PO_4_)_2_ was also detected. Due to the complexity of the bioglass-composition, XRD graphs could not be evaluated. The measured peaks could not be clearly assigned to the ceramic due to the complicated composition of the glass.

#### 3.1.3. Raman Spectroscopy Phase Composition Determination of the Metals

All Raman spectroscopy data can be found in the Supplementary Material (Figures S6–S12). Raman spectroscopic analysis of the bioglass + Cu layer revealed the presence of copper as a pure metal, but also copper phosphate Cu_3_(PO_4_)_2_, tenorite (CuO) and cuprite (Cu_2_O). Silver oxide Ag_2_O and silver phosphate Ag_2_PO_4_- could be detected in the bioglass + Ag layer. Bismuthinite (Bi_3_S_3_) could be detected in the bioglass + Bi layer. In the GB14 + Cu layer copper could be detected in its metallic form, in GB14 + Bi layer α-Bi_2_O_3_ could be detected. In GB14 + Ag coating the crystalline layer was slightly yellowish but no silver-containing substance was identifiable. In the HA + Ag layer silver phosphate Ag_3_PO_4_ could be identified. α- Bi_2_O_3_ were detectable in the HA + Bi layer composite. Copper phosphate Cu_3_(PO_4_)_2_ and copper nitrate Cu(NO_3_)_2_ were detectable in the TCP + Cu layer. In the TCP + Ag layer only black shining crystals were visible, which did not show any other compound in the Raman spectrum apart from TCP. The crystals were probably metallic silver. Whether these crystals were formed by photolysis or chemically cannot be determined by Raman measurements. In TCP + Bi no colored crystals could be found. The coating seemed to consist exclusively of amorphous and crystalline TCP. 

#### 3.1.4. SEM for Verification of Metal Doping Content of the Coatings

**Figure 3:**
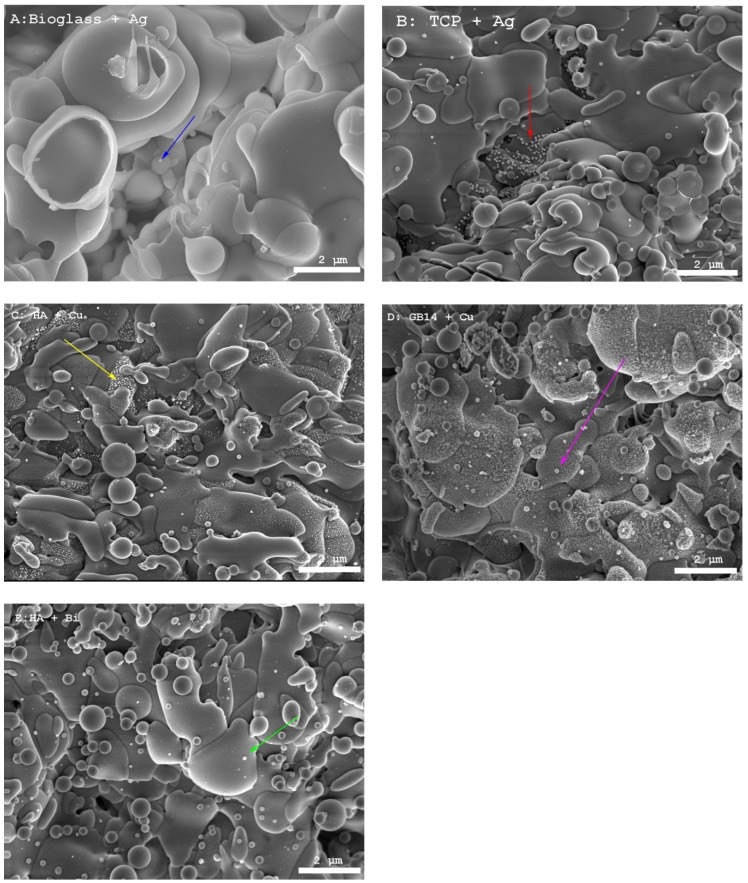
Selection of SEM images of the coatings, 5000-fold magnification, arrows showing metal particles, (**A**) bioglass with silver; (**B**) TCP with silver; (**C**) HA with copper (**D**) GB14 with copper; (**E**) HA bismuth.

The SEM images of the various coatings (Figure 3A–E, supplement Figure S13) show typical features of thermally sprayed surfaces: spray droplets and splashes as well as spherically resolidified particles can be detected. As can be seen in the pictures in Figure 3 the microstructure of the different Ca-phosphates have a comparable structure. 

The SEM images also served as visual proof of the metal doping in the ceramic layers. Two phases could be distinguished: on the one hand the spray droplets and on the other hand an additional phase which had deposited between the droplets. The two phases could clearly be distinguished on the silver-doped layers (see red arrows in Figure 3B).

On bismuth-doped layers the bismuth phase appeared to have settled superficially on the coating (Figure 3E, green arrow). The copper in the copper-doped layers appeared to have a coarser structure than the other metals, thus it was less clearly distinguishable from the ceramic droplets (brown arrow Figure 3D). On the bioglass coatings, the quantity of visible metal phase showed to be less than on the other ceramics (see blue arrow in Figure 3).

#### 3.1.5. Element-Mapping for Cu-Content Verification 

**Figure 4:**
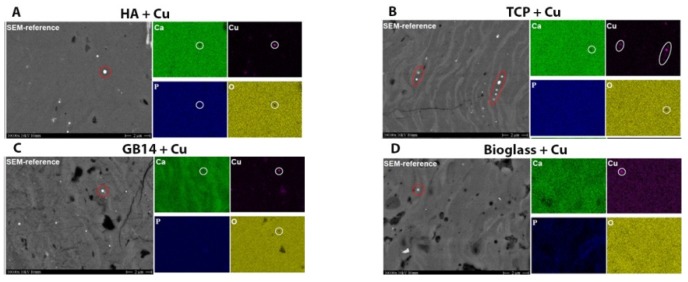
Element-maps of the copper-supplemented coatings. (**A**) HA with Cu; (**B**) TCP with Cu; (**C**) GB14 with Cu; (**D**) Bioglass with Cu. Ca: Calcium weighed, Cu: copper weighed, P: Phosphate weighed O: oxygen weighed cuts. The images were taken at 3 kV with a SEM Zeiss DSM 982 Gemini Microscope at the IFKB Stuttgart.

The IMTCCC produced element maps of polished cuts of Cu-doped. The element-map analysis was limited to Cu-doped layers for cost reasons. A scanning electron microscope control image of the samples was taken as a reference, in which the brighter dots (Figure 4 circled in red) correspond to the bright spot in the Cu-weighted image (Figure 4 Cu, circled in white). For Cu particles >300 µm a gap in the oxygen-weighted image (Figure 4 O) and in the Ca-weighted image (Figure 4 Ca) was detected, indicating the presence of metallic copper. Due to the limited resolution of the element mapping, copper oxide or metallic copper could not be distinguished in smaller particles [38].

#### 3.1.6. Release Kinetics of Coating Contained Metal Ions and Ceramic Components into Water

The exact values of the released metal-ion quantity can be found in the Supplementary Material (Tables S3 and S4). The quantity of metal released did not exceed a maximum of 0.014 µg per mL H_2_O. This maximum was reached by GB14 + Cu after 72 h. For all other samples the values were below 0.01 µg/mL H_2_O or in a non-measurable range. All time points put together the GB14 + Cu-, GB14 + Bi- and the bioglass + Cu-coatings showed the highest metal ion release values. In the water-analysis for ceramic components only Ca^2+^ and PO_4_^3−^ ion were found to be in a measurable range. The maximum release values for Ca^2+^ were reached by HA + Cu (0.22 mmol/L) and GB14 + Ag (0.23 mmol/L) after 6 h and for bioglass + Bi after 72 h. PO_4_^3−^ ions could be measured in a concentration of 0.1 mmol/L for bioglass + Cu, bioglass + Ag, GB14 + Cu, HA + Ag and TCP + Bi at all times. The exact values can be found in the Supplementart Material. 

### 3.2. Biocompatibility

#### 3.2.1. Live/Dead-Assay and Cell Count

In addition to the display of the cells attached to the coating the live/dead assay allows to differentiate living from dead cells on the surface of the coatings. Under the fluorescence microscope (Olympus BX-51) dead cells appear red, living cells appear green. 

**Figure 5:**
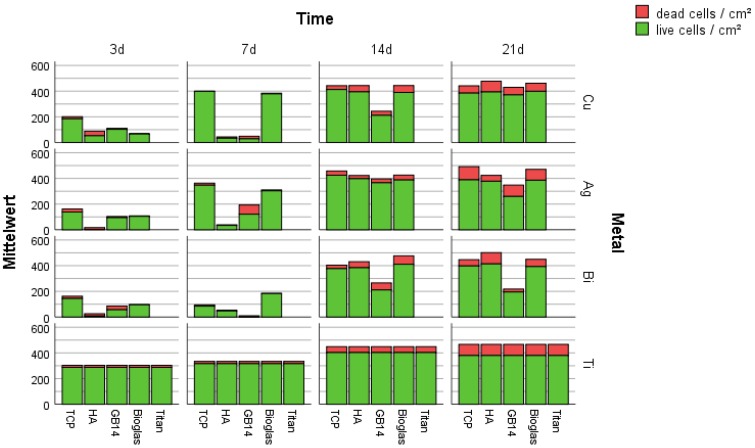
Dead/live assay. Cell number (live and dead) on the metal doped hydroxylapatite, tricalcium phosphate, GB14 and bioglass coatings after 3, 7, 14, 21 days.

**Figure 6:**
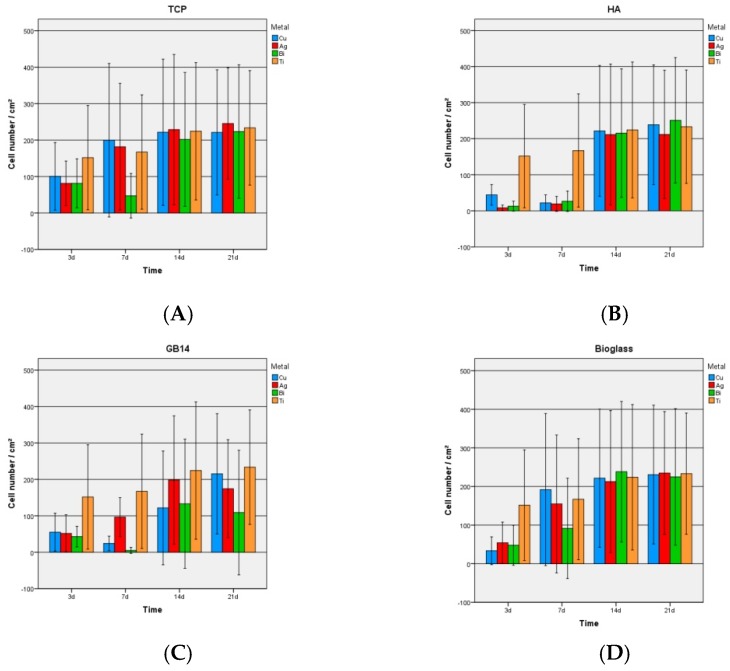
Cell numbers on the coatings. After 3, 7, 14 and 21 days. (**A**) TCP (**B**) HA (**C**) GB14 (**D**) bioglas.

The pictures of the Live/Dead assay show that on most of the samples cells reached confluence after at latest 21 days (supplement Figure S14). On HA + Cu a dense cell layer had formed only on day 14 (supplement Figure S14). On GB14 coatings only a few cells were visible on the surfaces until day 14 (supplement Figure S14). The cell counts (Figure 6) showed that the cell number of cells increased over time (Figure 5 and Figure 6). Cell growth on the titanium control, the bioglass and TCP coatings regardless of the metal doping was comparable: after 7 days a dense cell layer with confluent cells could be found (Figure 5). On the mentioned coatings, the number of dead cells in proportion to the total cell count remained low. On hydroxyapatite fewer vital cells and relatively more dead cells were visible at early points of incubation time (Figure 5). On HA coatings the state of a homogeneous cell-layer was only reached on day 14. Cell growth on the GB14 coatings was missing completely at some points in time; only on GB14 + Ag the cell-growth was comparable to the titanium control. On GB14 + Cu/Bi the cells behaved unpredictably: at sometimes they were completely missing on the coating, or they were only found in small groups (Figure 5 and Figure 6).

#### 3.2.2. Proliferation

**Figure 7:**
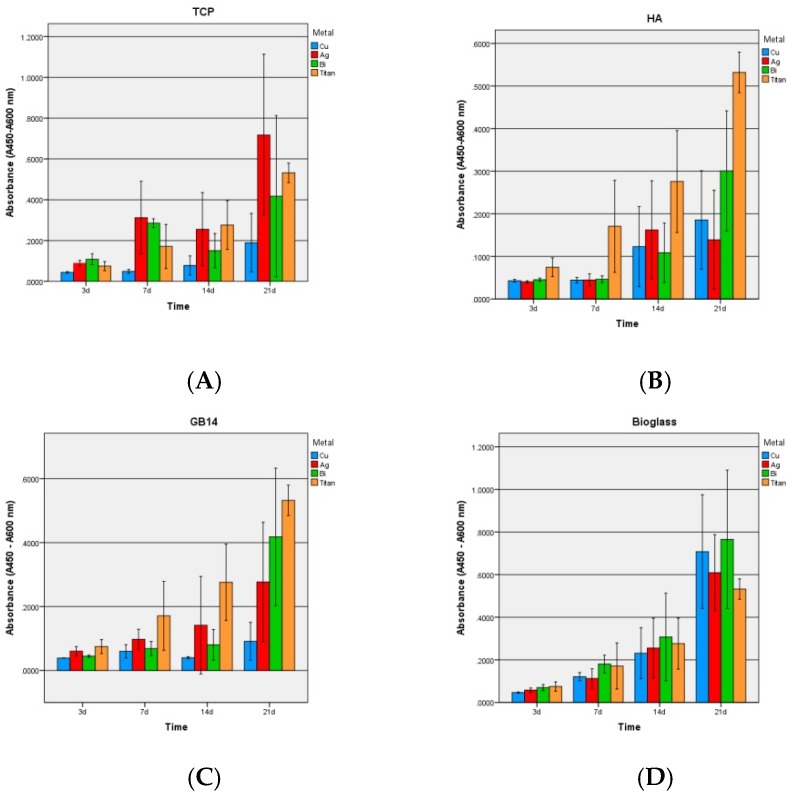
WST-1: Cell vitality of the coatings over time after 3, 7, 14, 21 days. (**A**) TCP, (**B**) HA, (**C**) GB14, (**D**) bioglass.

Overall, TCP + Ag and the bioglass coatings (Figure 7A,D) showed the highest vitality values and the most constant cell growth over the examinated period of time, comparable to the cell growth on the titan-control. GB14 coatings presented few cells at all times (Figure 7C), especially at the early incubation times.

On the HA coatings (Figure 7B) no cell growth and no cell increase was observed between day 3 and 7, cell count then approached the level of TCP and bioglass coatings on day 14. With regard to doping, TCP + Ag and all bioglass coatings in particular showed high cell counts. Copper-containing ceramics showed a lower WST-1 proliferation overall.

#### 3.2.3. LDH-Kit

**Figure 8:**
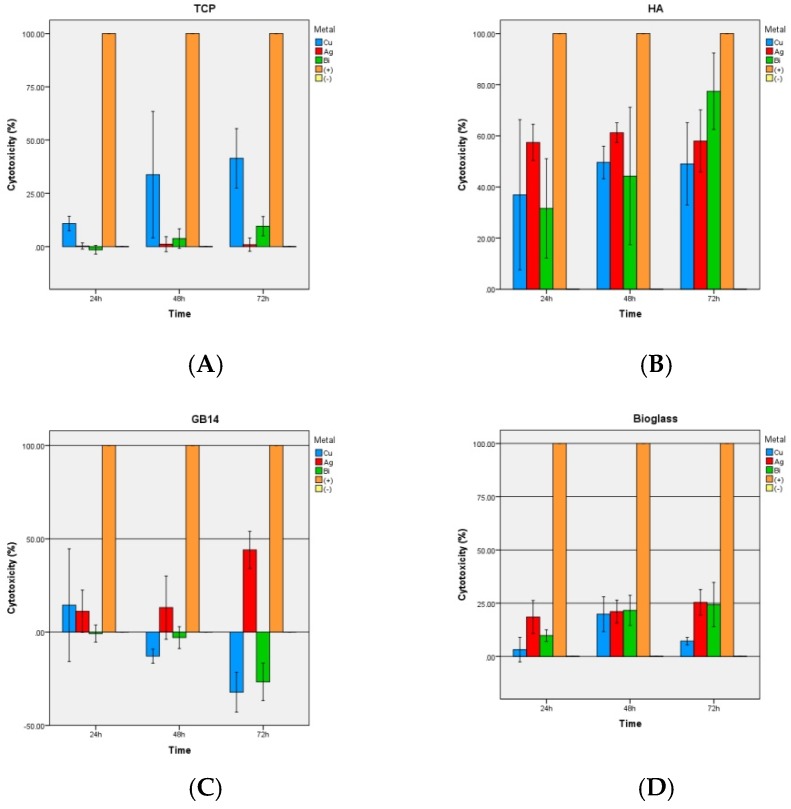
LDH: Cytotoxicity of the ceramic groups after 3, 7, 14, 21 days. (**A**) TCP, (**B**) HA, (**C**) GB14, (**D**) bioglass. (+) Positive control (-) Negative control.

Overall, the HA coatings regardless of the metal showed significantly higher toxicity values than the other coatings (Figure 8B).

A comparatively significant low cytotoxicity was found in TCP and bioglas coatings (Figure 8A,C), though the copper doped TCP coating exhibited significantly higher cytotoxicity than TCP + Bi/Ag. GB14 coatings had the lowest toxicity values (Figure 8C).

#### 3.2.4. SBF-Experiment

**Figure 9:**
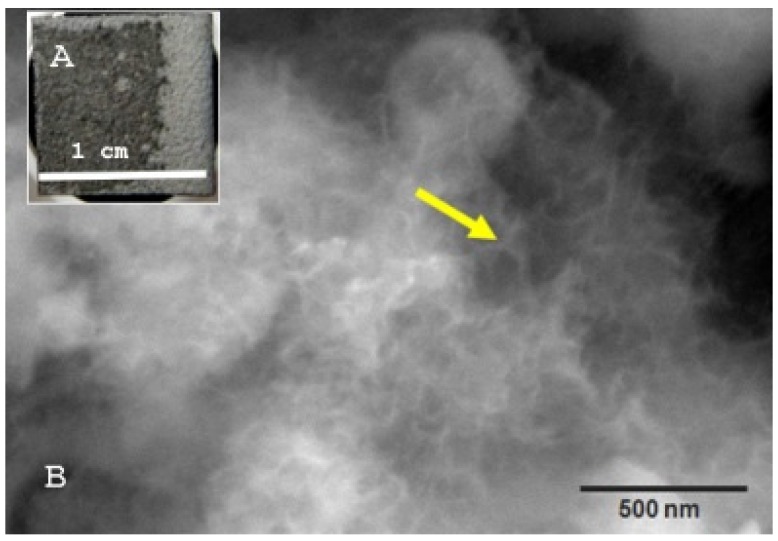
TCP + Ag Coating: (**A**): macroscopic image of the sample after 28 d incubation in SBF, (**B**): Scanning electron microscope image, 130,000× magnification. Yellow arrow points to a HA crystal.

**Figure 10:**
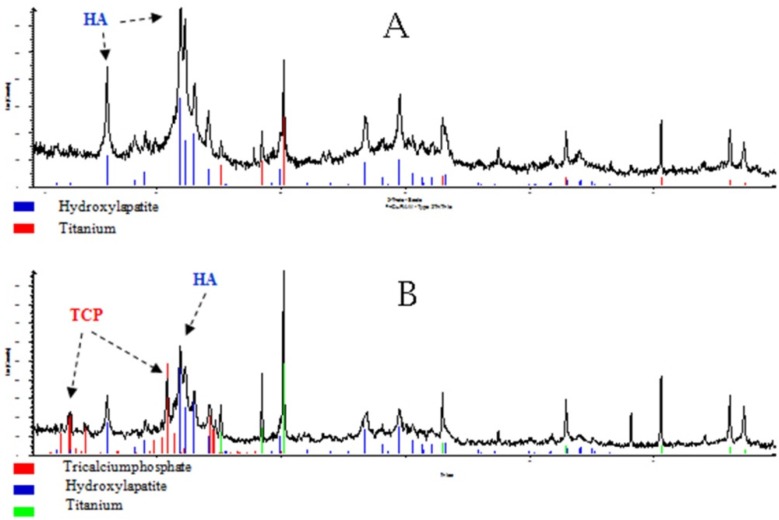
XRD-Analysis of the HA + Cu (**A**) and the ß TCP + Ag (**B**) coatings after 28 days in SBF.

After 28 days in SBF, macroscopic colour and structural changes on the sample surface could already be detected, as shown here for TCP + Ag (Figure 9A). Electron microscopically, crystal structures could be detected especially on TCP + Ag and HA + Cu at 120,000 and 130,000 times magnification (yellow arrow Figure 9B). GB14 + Bi crystals could only be displayed at 50,000 magnification. On TCP + Cu and Bioglas + Bi the crystals could not be seen microscopically. The fact that the crystals seen on the coatings were constituted of hydroxyapatite was confirmed by XRD analysis of the TCP + Ag and HA+ Cu sample (Figure 10).

### 3.3. Antimicrobial Testing with Safe Airborne Antibacterial Assay 

The Airborne Antimicrobial Assay was conducted to evaluate the antimicrobial effects of the different doped coatings. Titanium served negative control for a surface without any antimicrobial effects. The less sprayed bacteria survived on the coating surface, the more antimicrobial effects were shown for this coating. 

**Figure 11:**
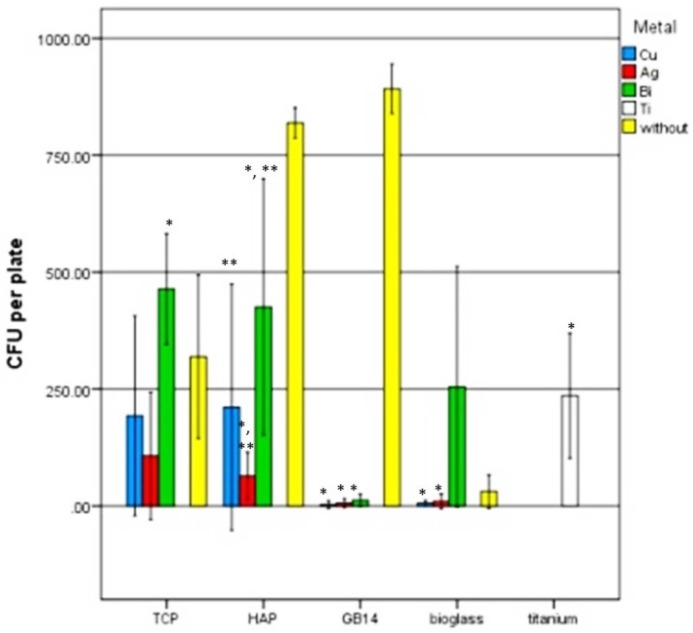
The number of colony forming units (CFUs per cm^2^) after an incubation time of the sprayed bacteria for 30 min. All values are triplicates with three replicates each. There are significant differences between coatings with metals and without metals A * symbol represents significant differences from titanium, A ** symbol significant differences between metals in the coating.

As shown in Figure 11, in comparison to titanium significantly less bacteria (the lowest CFU value, *p* = 0.021) survived on the silver-doped coating of tricalcium phosphate (TCP), whereas even significantly more (*p* = 0.001) CFU could be detected from the TCP-coating doped with bismuth. No significant differences (*p* > 0.05) of the survived CFU were shown between titanium and the TCP-coating doped with cupper. Similar results were shown for hydroxyapatite (HA) coating. The silver doped HA surface showed significantly higher (*p* = 0.002) antimicrobial effect (lower CFU value) as compared to titanium. The bismuth-doped HA coating showed significantly higher (*p* = CFU values than the silver- (*p* < 0.001) or copper (*p* = 0.040)-doped HA coating. Doped glass ceramic (GB14) coating led to significantly less CFU value (*p* < 0.001) in comparison to titanium independently of the metal used. Doping bioglass coating with silver or copper led also to a significantly lower CFU value (*p* < 0.001) on the surface as compared with titanium, whereas the bismuth-doped bioglass coating showed a similar CFU value (*p* > 0.05) as titanium. Pure coatings without metals lead to high CFU values in comparison to coatings with Cu and Ag. It should be considered that bioglass has certain antibacterial activity per se. 

It appeared that the highest antimicrobial potency, that means the lowest CFU formation, could be found on the silver-doped coatings no matter which ceramic component it was combined with (Figure 11). Also copper, especially in combination with bioglass and β-TCP showed to be highly effective. Those two metals in combination with GB14 and bioglass ceramics showed the highest antimicrobial potency. The lowest antimicrobial effect of the metal doping was found on the bismuth-containing coatings: in combination with every ceramic, Bismuth-doped coatings allowed higher CFU growth compared to the other layers.

## 4. Discussion

The colonization of biomaterials with bacteria represents the main cause of implant-associated infections. Both an antibiotic prophylaxis and a faster osteointegration can be obtained by incorperation of bactericidal active metals in degradable CaP coatings. At present there is no reliable method on the basis of thermal spraying to get thin homogeneous layers containing silver, copper and bismuth in bacteriostatic/bactericidal concentrations. With the help of high-velocity suspension flame spraying (HVSFS) process it is possible to produce such thin resorbable bioactive ceramics coatings on the basis of degradable calcium phosphates and bioactive glasses. In these layers bacteriostatic/bactericidal effective metals silver, copper and bismuth were integrated. The aim of this study was to evaluate in vitro biocompatibility and antimicrobial activity of the coatings. The tests carried out revealed that the coatings had different biocompatibility on MG-63 cells and did not have identical antimicrobial efficacy on *S. aureus*. 

### 4.1. Live/Dead-Assay

With the help of the viability studies, it is possible to assess the biocompatibility of materials for bone replacement. The coatings contain small amounts of metals with bactericidal activity. During the degradation of the coating, ions are released that can prevent biofilm formation. The degradation of the coating takes much longer. The process should be completed after half a year. This is the only way to achieve faster osseointegration (degradable coating) combined with infection prophylaxis.

The Live/Dead assay showed that on most of the samples cells reached confluence after at latest 21 days. While the cell growth on bioglass and β-TCP did show to be as constant as on the titan control throughout the experiment, the cell growth on HA an GB14 did show unexpected variations.

On HA-coatings fewer vital cells and relatively more dead cells were visible at early points of incubation time. On HA coatings the state of a homogeneous cell-layer was only reached on day 14 instead of day 7 as for other coatings. 

Hydroxyapatite as such is known to be a biocompatible and osteoconductive material [39]. However, in some forms for example as mortar granules with particles of different sizes [40] or powders with particle sizes of 0.5 to 841 µm [41] it can prove to be cell toxic. In vitro experiments have shown that the size of HA particles determines its cell effect. Sun et al. [41] and Evans et al. [40] came to the conclusion that particularly small hydroxyapatite particles are phagocytized by osteo- and fibroblasts and thus damage the cell. In addition, Ewence et al. [42] could show that calcium phosphate crystals released from HA induce apoptosis in myo- and fibroblasts. It is possible that such small hydroxyapatite particles were released from the HA samples and had a cell-toxic effect. But this explanation does not sufficiently justify why the HA layers seemed to show a high cytotoxicity especially at the early incubation times (3, 7 days), while the growth at the later stages (14, 21 days) was similar to the growth on the titan-control. Eventually this phenomenon could be due to the fact that a cell-toxic substance was produced during the spray-process of the coatings and released from the coatings over time during the experiment. The substance being released decreasingly, it spread into the surrounding medium and was removed from the wellplates with every medium change, until none of the toxic substance was longer contained whether in the coating nor in the medium. 

The formation of toxic substances could be explained by the oxidation of certain coating elements. In fact, an oxygen/ethene mixture was used as fuel gas for the layer production process. The oxidation of ethylene leads to the formation of ethylene oxide (C_2_H_4_O), the simplest epoxide. Since oxygen and ethylene oxide (EO) are also strong reducing agents, an undesired reaction with elements of the HA layer (Ca_5_(PO_4_)_3_(OH)) is conceivable due to the high process temperatures (2900 °C). For example monophosphane (phospine, PH_3_) could have been formed by reduction of the phosphate group in hydroxyapatite [43]. Epoxides and phosphines have strong cell toxic effects: Ethylene oxide (EO) is used for sterilization of various materials (textiles, medical supplies, etc.) because of its antimicrobial, antiviral and antifungal effects [44,45]). These effects are caused by the alkylation of DNA and RNA strands of organisms by the EO, which irreversibly inhibits cellular metabolism [46]. Tan et al. [47] demonstrated the mutagenicity and cytotoxicity of EO in Chinese hamster overial cells. Phospins are used in agriculture as insecticide and fungicide [48,49]. The cytotoxic effect of phosphines is caused by inhibition of cytochrome c oxidase, which hinders the mitochondrial respiratory chain [50]. On the cellular level, Rashedina et al. [51] could detect the cytoxicity of phosphines on HepG2 cells.

That the substances produced during the spray process and not the hydroxyapatite itself had a cytotoxic effect could be confirmed by further experiments of our working group: In these experiments the cell vitality on unwashed HA samples was compared with the cell vitality on washed (72 h at 200 rpm in distilled water) HA samples. On washed samples toxicity was no longer detectable, as the samples had been freed from the toxins during the cleaning process.

Also on GB14 coatings the cell growth did not show to run as smooth as on the titan control, bioglass and TCP coatings. Only few cells were visible on the GB14-coated surfaces until day 14 only on GB14 + Ag the cell-growth was comparable to the growth on the titanium control. On GB14 + Cu/Bi the cells behaved unpredictably: at some incubation times cells were completely dismissed on the coating or they were only found in small groups.

Toxic effects of solved GB14 ion release have been described in literature [52], but the low solubility product of GB14 could also be an explanation for the above mentioned results. Berger et al. [53] could prove that GB14 is five times more soluble than α-TCP, which has a similar biodegrability as β-TCP [54]. Coatings thus may have dissolved quickly into the surrounding fluid after having been seeded with cells and placed into wellplates with the default medium. The cells originally attached to the coating could that way have spread into the environing fluid and been pipetted up with every medium change. As a result no or few cells could be seen through fluorescence microscopy at early incubation times. The remaining cells contained in the well could then have attached to the samples without the coating and have proliferated to finally build a dense cell layer as observed in the live/dead assay at later incubation times.

### 4.2. WST-1 Assay

In the WST-1-Experiment TCP and the bioglass coatings showed the highest vitality values and the most constant cell growth over the examined period of time, comparable to the cell growth on the titanium control. GB14 coatings presented few vitality rates at all times, especially at early incubation times. On the HA coatings no cell growth and no cell increase was observed between day 3 and 7, the vitality rate then approached the level of TCP and bioglass coatings on day 14. These results are congruent with the ones seen in the dead-live assay and here again, the toxicity of the HA coatings at early incubation times could be due to the fact that a cell-toxic substance was produced during the spraying-process and was released from the coatings. The low values for GB14 could also like in the Live/Dead assay be due to the fact that the coating dissolved into the surrounding medium and took cells away with it. 

With regard to the doping silver- and bismuth-containing coatings showed higher WST-proliferation than copper-containing ceramics. This phenomenon had not been observed in the Live/Dead assay. An explanation for these results could be the contained copper amount in the coatings which could have cell-toxic effects. The coatings examined here contained 9.825 mg ceramic and 0.175 mg copper acetate, that means 3.37 × 10^−7^ mol Cu^2+^ ions. Not all metal contained in the coating could come into direct contact with the cells, since the metal ions were released according to the solubility of the ceramic they were integrated in. Accordingly, the cells on GB14 and bioglass must have come into contact with the released metals earlier than the cells on TCP and HA since the first mentioned ceramics have a higher solubility. In the release kinetics experiment the maximal copper-ion release was found in bioglass + Cu with a total Cu release of 0.032 µg/L, i.e. an equivalent of 5.03 × 10^−8^ mmol/L. This value does not come close to the cytotoxic copper values found in literature [55,56,57].

An explanation for the irregularity of the results regarding to the copper-content of the coatings could be an interaction of the WST-1 kit elements with the metal contained in the coatings. In the WST-1 kit tetrazolium salt (3-(4,5-dimethylthiazol-2-yl)-2,5-diphenyltetrazolium bromide) is transformed into formazan by mitochondrial dehydrogenases of metabolically active cells. The arisen formazan can then be quantified photometrically depending on the strength of the coloration. The more vital cells there are, the more formazan is produced and the measured absorption increases. 

Some working groups which investigated the cell viability with respect to copper-containing samples using the WST-1 kit found no increased cell toxicity of the Cu samples compared to the control [58,59,60]. On the other hand Semisch et al. [61] described an interaction of CuO and CuCl_2_ with the WST-8 kit. The functionality of the WST-8 kit is based on the same principle as that of the WST-1 kit: The reduction of tetrazolium salt leads to colored formazan. However, the WST-8 is more sensitive than the WST-1 test [62]. Nevertheless an interaction of the copper ions contained in our samples is therefore quite conceivable, since the copper elements could have interacted with the NADH + H^+^ during the reduction of the formazan.

### 4.3. LDH-Assay

Also the LDH assay results showed similarity to the other biocompatibility experiments. HA coatings had significantly higher toxicity values than the other coatings, regardless of the metal doping. A comparatively low cytotoxicity was found in TCP and bioglass coatings. 

The copper-doped TCP coating showed significantly higher cytotoxicity than TCP + Bi/Ag coatings. GB14 coatings had the lowest toxicity values. The above given explanations (4.2) concerning the low HA and GB14 values can be taken as a reason for the congruent results of the LDH-assay. The higher toxicity of the copper-doped TCP sample could again be due to an interaction of the metal with the kit: As in the WST-1 Kit in the LDH test, lactate dehydrogenase of damaged cells (LDH) reduces NAD^+^ to NAHDH^+^ + H^+^ by oxidation and lactate becomes pyruvate. In a second reaction, 2H of the NADH + H^+^ is transferred to the yellow tetrazolium salt by a catalyst. The color of the resulting formazan can then be quantified by absorption measurement. The higher the absorbance, the more damaged cells are contained in the well. 

Karlsson et al [63] described the impossibility of using the kit for Cu-containing samples due to an interaction of Cu particles with the assay. They demonstrated a clear increase in “LDH absorbance” when Cu, CuO, CuZn nanoparticles were present in the well. Cu nanoparticles could catalyze the conversion of NADH + H^+^ into NAD^+^ even in absence of LDH. Another reason why the wished chemical reaction was inactivated would be the absorption of LDH at the cell surface by the Cu particles, as described by Han et al. [64]. 

### 4.4. SBF

According to Kokubo et al. [65], materials that allow the formation of HA crystals on their surface are more likely to also allow this process in vivo. The resulting secondary HA layer on the surface induces the bone to grow closer to the material. The SBF method is therefore used for screening and as an orientation to determine which materials should be used for further investigation in animal experiments [65,66]. In our experiment HA crystal formation was found to be particularly pronounced on TCP, GB14 and HA after 28 days. The crystals on the mentioned samples had a similar aspect, a comparable crystal morphology and were found on the samples in similar densities. The formation of HA crystals had already been described on all examined ceramics: on HA, GB14, [9], bioglass [67], and TCP layers [68]. According to Stiegler et al. [69], crystal formation on HA coatings depends on the microstructure of the coating, for example coatings with a low crystallinity dissolve faster in SBF then rapidly re-precipitate on the surface and form crystals. The formation of HA crystals on surfaces after incubation in SBF is considered to be a positive predictor for good osteoconduction in vivo [65,70,71]. However, the results obtained in vitro can only be transferred on a living organism to a limited extent. Certain influences, such as the influence of the musculoskeletal system on the growth of bone and the coated implant cannot be observed in vitro [72,73].

### 4.5. Safe Airborne Antibacterial Assay

The results of this experiment showed the same tendencies in all three replicates: the highest antimicrobial potency on *Staphylococcus aureus* among the metals was attributed to silver. Bismuth had the lowest antimicrobial effect. As for the ceramics, the bioglass and GB14 layers seemed to potentiate the effect of the metal doping. The higher solubility of certain ceramics and therefore the quicker release of antibacterial active metal ions could also explain why GB14 and bioglass had the highest solubility among the examined ceramic-coatings. It is therefore conceivable that more metal ions were released in larger quantities from GB14- and bioglass-constituted coatings, and could that way show a stronger antimicrobial effect. It is also described in literature that bioactive glasses themselves have antibacterial properties in vitro, due to ion exchange processes with the surrounding medium that increase the medium pH [52,74], this could also explain the particularly strong antimicrobial effect of bioglass coatings.

It’s been noticed that bismuth had weak antimicrobial effects in this experiment, although bismuth is known for its antibacterial virtues and bismuth compounds are therefore used in many areas of medicine: bismuth salt is used against gastrointestinal inflammation, particularly for the eradication of *Helicobacter pylori* (in combination with a proton pump inhibitor, tetracycline and metronidazole) [75]. In this work, antimicrobial effects were tested on *Staphylococcus aureus*. A reason why bismuth did not show strong antimicrobial effects on *S. aureus* could be the resistance of this strain to bismuth ions. In fact Novick et al. [76] found that penicillinase plasmids in *S. aureus* can cause resistance of the bacterium to various metal ions, especially bismuth. This finding was later confirmed by other working groups [77]. The resistance mechanism is the same as the one for penicillin resistance of *S. aureus* and could even favor the latter [78]. 

## 5. Conclusions

In this work the biocompatibility and antimicrobial virtues of 12 silver-, copper- or bismuth- containing ceramic coatings were examined in order to determine the ceramic/metal combination with the most suitable properties.

In terms of biocompatibility on MG-63 osteoblast-like cells, β-TCP and bioglass coatings showed the highest cell compatibility and the lowest cytotoxicity (dead-live assay, WST-1, LDH). The biocompatibility of GB14 could not be classified reliably due to its high solubility and the resulting difficulty of cell adhesion to the samples surface. HA showed a lower biocompatibility compared to the other ceramics at the beginning of the experiments which could have been caused by the release of toxic substances at early incubation times (3 days, 7 days). This was confirmed by the fact that the same HA samples washed with distilled water earlier and then used for cell viability tests did not show any toxicity. Metal doping had a significant influence on the antimicrobial effect: while Ag and Cu-doped layer composites showed strong antimicrobial activity, Bi-doped layers were ineffective, probably due to a resistance mechanism of *S. aureus* bacteria. GB14 and bioglass ceramics potentiated the antimicrobial Ag and Cu effect, on the one hand through a high ion release from the composites, on the other hand through bioglass in itself by being known for its antimicrobial virtues.

In conclusion the best biocompatibility and simultaneous strong antimicrobial effect could be assigned to the bioglass + Ag and bioglass + Cu coatings, followed by the GB14 + Ag and GB14 + Cu coatings. With the help of high-velocity suspension flame spraying (HVSFS) process it is possible to produce biocompatible thin resorbable ceramics coatings containing effective bacteriostatic/ bactericidal metals. These coatings can accelerate integration into the bone on the one hand and prevent biofilm formation on the other.

## Figures and Tables

**Table 1:** Overview of the ceramics used.

Ceramic	Chemical Composition	Particle Size (µm)	Manufacturer
Hydroxyapatite	Ca_10_(PO_4_)_6_(OH)_2_	5.8	CeramTec GmbH, Plochingen, Germany
β-Tricalcium phosphate	Ca_3_(PO_4_)_2_	3.8	Chemische Fabrik, Budenheim, Germany
GB14	Ca_2_KNa(PO_4_)_2_	17.4	Bundesanstalt für Materialforschung und—Prüfung (BAM), Berlin, Germany
Bioglass	47.3% SiO_2_, 28.6% CaO, 15.2% P_2_O_5_, 4.90% Na_2_O, 2.50% MgO 1.5% F	8.0	Centro Ricerche Colorabbia Consulting, Florence, Italy

**Table 2:** Suspension-composition for the coatings.

Coating	Solids Content (wt.%)	Metal Content (Mass) Related on Solids Content (%)	Liquid Phase
HA + Cu	10	1.75	Iso/H_2_O
HA + Ag	10	1.75	Iso/H_2_O
HA + Bi	10	1.75	Iso
TCP + Cu	10	1.75	Iso/H_2_O
TCP + Ag	10	1.75	Iso/H_2_O
TCP + Bi	10	1.75	Iso
GB14 + Cu	10	1.75	H_2_O
GB14 + Ag	10	1.75	H_2_O
GB14 + Bi	10	1.75	H_2_O
Bioglass + Cu	10	1.75	Ethanol
Bioglass + Ag	10	1.75	Ethanol
Bioglass + Bi	10	1.75	Ethanol
Iso = Isopropanol, H_2_O = Water			

**Table 3:** Parameters for Raman spectroscopy.

**Laser (nm)**	638, 532	**Number of Scans**	30–100
**Power (mW)**	2.5–12	**Aperture Time Per Scan (sec.)**	5
**Grid (gr/mm)**	1200	**Magnification (Microscope)**	10–50

**Table 4:** Quantities of pure metal for coating.

Cu(II) (from Cu(CH_3_COO)_2_)	6.12 × 10^−5^ g	3.37 × 10^−7^ mol
Ag (from AgNO_3_)	7.06 × 10^−5^ g	6.54 × 10^−7^ mol
Bi	1.75 × 10^−^^4^ g	8.40 × 10^−^^7^ mol

## Supplementary Materials

The following are available online at https://www.mdpi.com/1996-1944/12/21/3495/s1, Figure S1: Surface morphology using high-resolution Field Emission Gun, Figure S2: XRD analysis of HA-coatings, Figure S3: XRD analysis of TCP-coatings, Figure S4: XRD analysis of GB14- coatings, Figure S5: XRD analysis of bioglass-coatings, Figure S6: Raman spectrometry of the bioglass + Cu coating, Figure S7: Raman spektroscopy of the bioglass + Ag coating, Figure S8 Raman spektroscopy of the bioglass + Bi coating, Figure S9: Raman spectroscopy of the GB14 + Bi and GB14 + Cu- coatings, Figure S10: Ramanspektroscopy of the HA + Cu, HA + Ag coating., Figure S11: Ramanspectroscopy of the HA + Bi coating, Figure S12: Raman spectroscopy of the TCP + Cu coating, Figure S13: SEM- Images of the coatings, Figure S14 Dead/live assay, cell expansion on the metal doped hydroxylapatite, tricalciumphosphate, GB14 and bioglass coatings after 3, 7, 14, 21 days, 10× magnification. Living cells appear green, dead cells appear red, Table S1: Roughness values (µm), sorted by coating composition. Table S2: Layer thicknesses of the samples, Table S3 Metal ion release-kinetics values, Table S4: Ceramic release in mmol/L, after 6, 24, 48 and 72 h.
