# Supplementary material for: Thin Degradable Coatings for Optimization of Osteointegration Associated with Simultaneous Infection Prophylaxis
Source: Materials (Basel). 2019 Oct 25;12(21):3495. doi: 10.3390/ma12213495 (PMC6862457; doi:10.3390/ma12213495)
Supplement: Supplementary file 1 [file materials-12-03495-s001.docx]

Supplementary

Thin Degradable Coatings for Optimization of Osteointegration Associated with Simultaneous Infection Prophylaxis

Sophie Burtscher ^1^, Peter Krieg ^2^, Andreas Killinger ^2^, Ali Al-Ahmad ^3^, Michael Seidenstücker ^1^, Sergio Hernandez Latorre ^1^ and Anke Bernstein ^1,^*

1. Surface roughness of the coatings

**Table S1.** Roughness values (µm), sorted by coating composition.

| **Coating** | **Ra (µm)** | **SD** | **Beschichtung** | **Ra (µm)** | **SD** |
| --- | --- | --- | --- | --- | --- |
| **TCP + Cu** | 11.25 | 0.50 | **GB14 + Cu** | 11.75 | 0.96 |
| **TCP + Ag** | 15.50 | 1.29 | **GB14 + Ag** | 7.25 | 0.96 |
| **TCP + Bi** | 12.25 | 0.50 | **GB14 + Bi** | 7.75 | 0.96 |
| **HA + Cu** | 10.00 | 0.82 | **Bioglass + Cu** | 10.00 | 0.82 |
| **HA + Ag** | 10.50 | 0.58 | **Bioglass + Ag** | 10.25 | 0.50 |
| **HA + Bi** | 11.75 | 2.22 | **Bioglass + Bi** | 9.75 | 0.50 |
|  |  |  | **Titan** | 7.25 | 0.96 |

2. Layer thickness and images of the coatings measured through bright field-microscopy

**Table S2.** Layer thicknesses of the samples.

| Coating | | Layer Thickness  (µm) | SD (µm) |
| --- | --- | --- | --- |
|  | **Cu** | 20.2 | 1.59 |
| HA | **Bi** | 14.4 | 1.64 |
|  | **Ag** | 23.9 | 1.83 |
|  | **Cu** | 15.7 | 3.44 |
| TCP | **Bi** | 17.8 | 2.67 |
|  | **Ag** | 16.9 | 4.20 |
|  | **Cu** | 27.0 | 5.83 |
| GB14 | **Bi** | 29.7 | 3.24 |
|  | **Ag** | 23.7 | 3.28 |
|  | **Cu** | 10.2 | 1.15 |
| Bioglas | **Ag** | 10.4 | 2.20 |
|  | **Bi** | 12.0 | 2.40 |

| 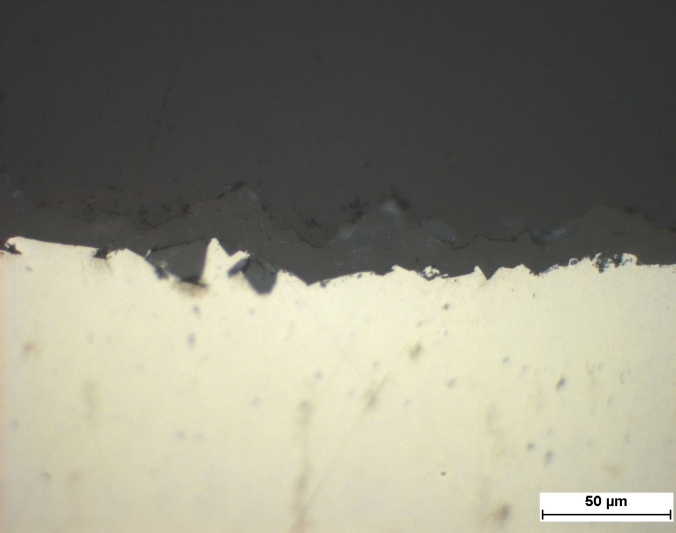  E  C  T  T  T  **a** | 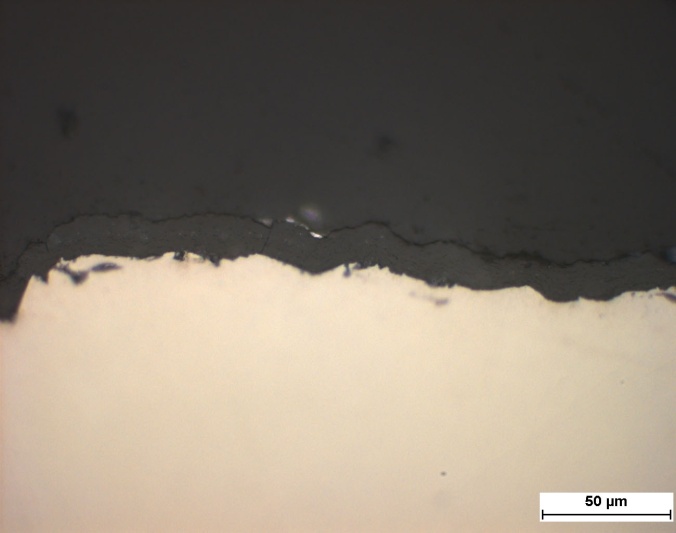  E  C  T  T  T  **b** |
| --- | --- |
| 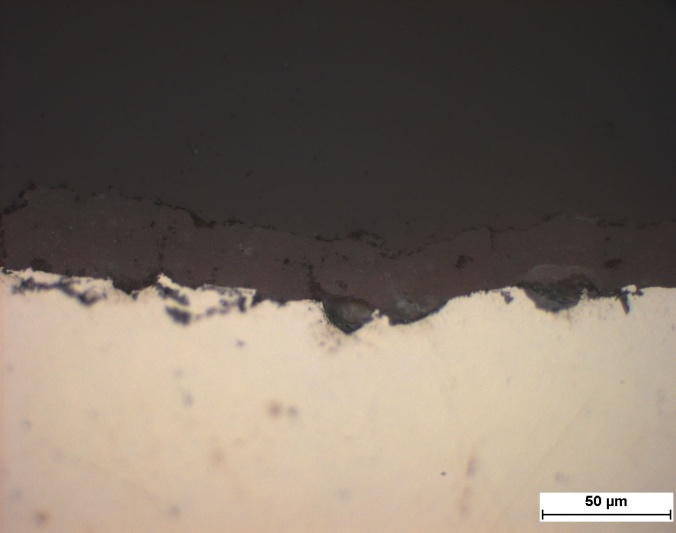  E  C  T  T  T  **c** | 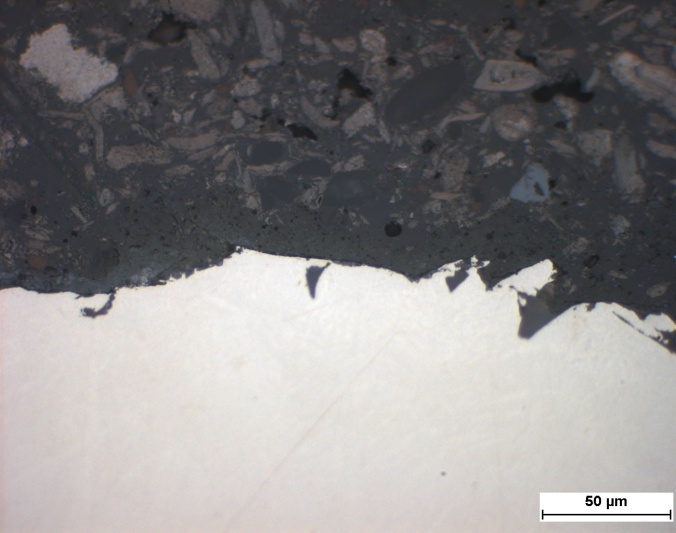  E  C  T  T  T  **d** |

**Figure S1.** Surface morphology using high-resolution Field Emission Gun. 500× magnification. **a**: TCP + Ag. **b**: HA + Bi. **c**: GB14 + Cu. **d**: Bioglass + Cu. T: titanium. C: coating. E: embedding material. The white bar corresponds 50 µm.

3. XRD measurements of the coatings


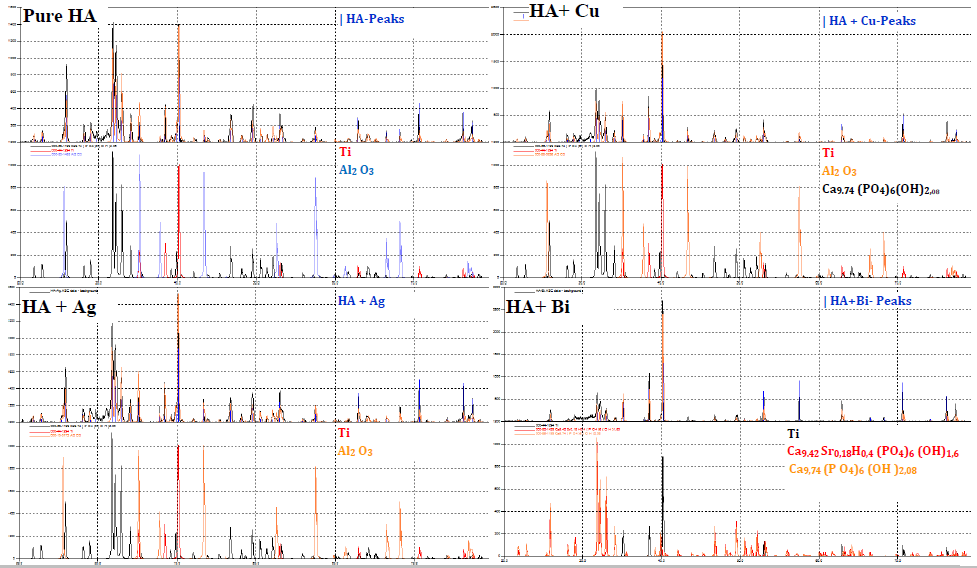


**Figure S2.** XRD analysis of HA-coatings.


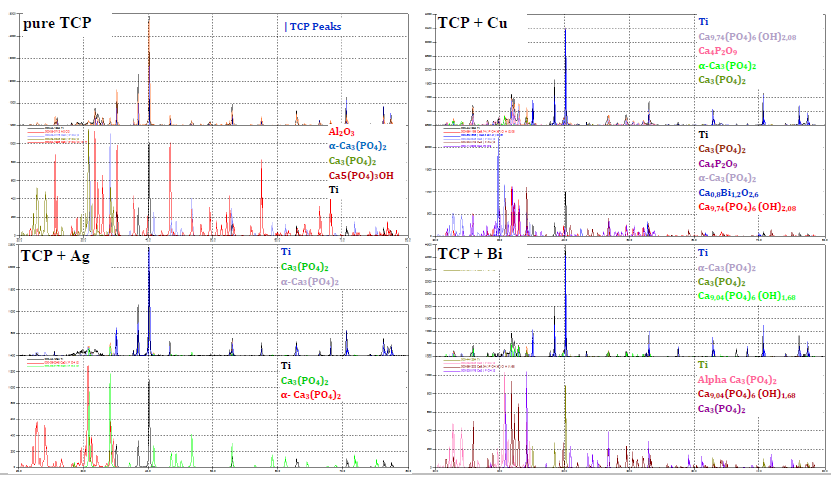


**Figure S3.** XRD analysis of TCP-coatings.


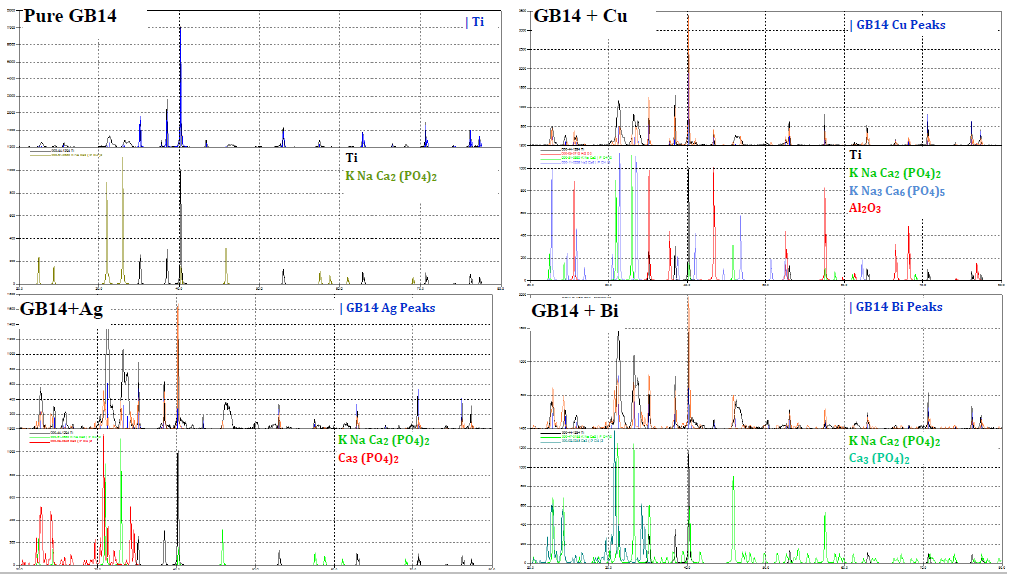


**Figure S4.** XRD analysis of GB14- coatings.


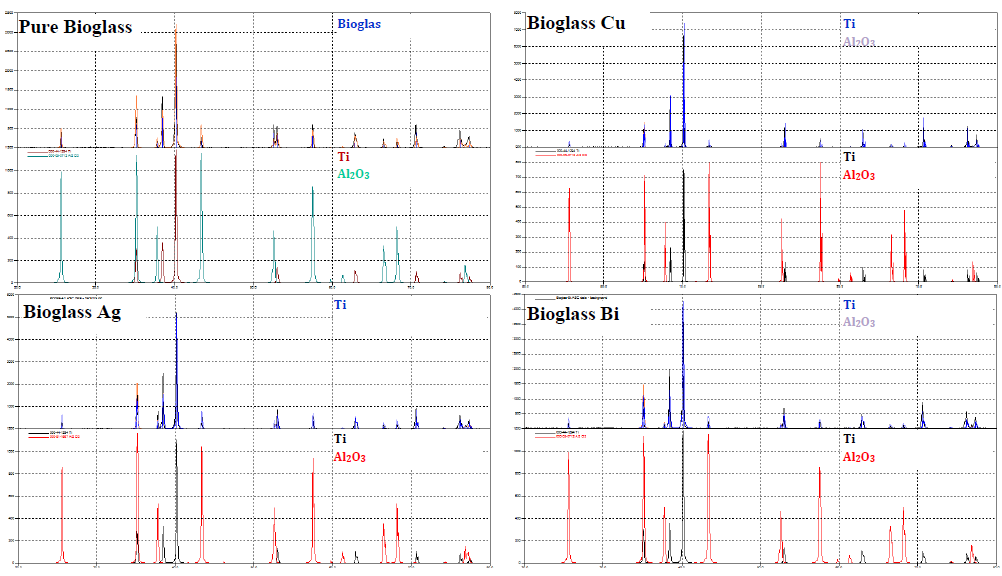


**Figure S5.** XRD analysis of bioglass-coatings.

4. Raman Spectrometry

| Picture of the Measuring Point | Associated Raman-Spectrum |
| --- | --- |
| Bioglass+ Cu |  |
| 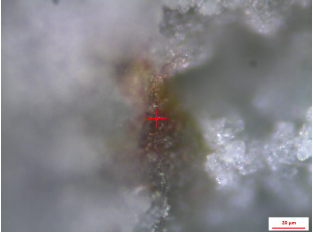 | 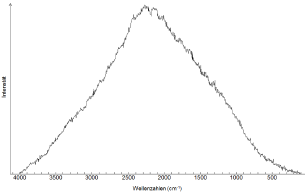  Cu Metall |
| 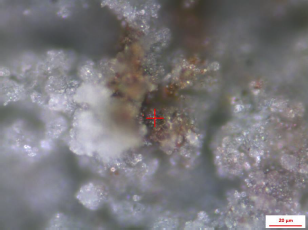 | 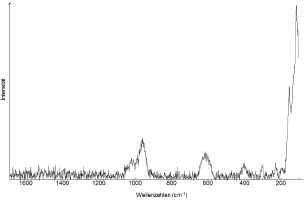  Cu_3_(PO_4_)_2_ mit wenig CuO |
| 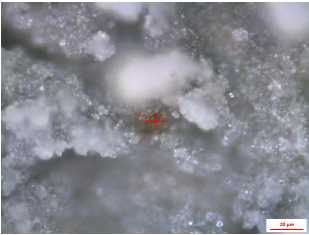 | 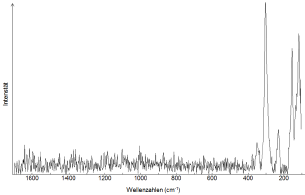 Cu_2_O mit CuO |

**Figure S6**. Raman spectrometry of the bioglass + Cu coating.

| Picture of the Measuring Point | | Associated Raman-Spectrum |
| --- | --- | --- |
| Bioglass + Ag |  | |
| 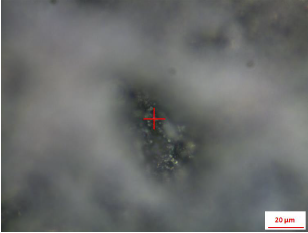 | 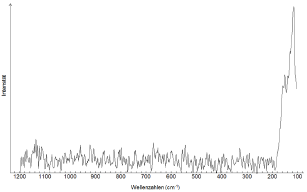  Ag_2_O | |
| 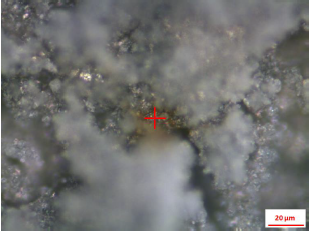 | 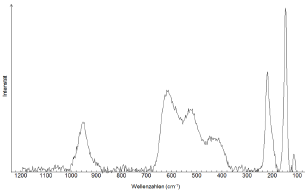Ag_2_PO^4-^ | |

**Figure S7.** Raman spektroscopy of the bioglass + Ag coating.

| Picture of the Measuring Point | | Associated Raman-Spectrum |
| --- | --- | --- |
| Bioglas + Bi |  | |
| 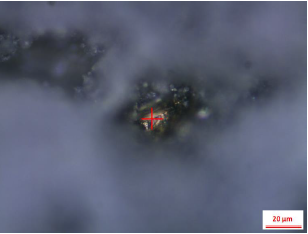 | 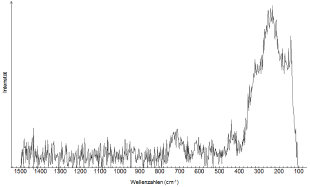Bi_3_S_3_ | |

**Figure S8.** Raman spektroscopy of the bioglass + Bi coating.

| Picture of the Measuring Point | | Associated Raman-Spectrum |
| --- | --- | --- |
| GB14+ Cu |  | |
| 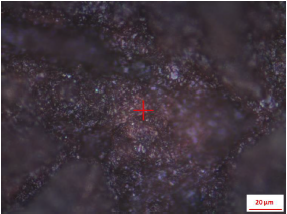 | 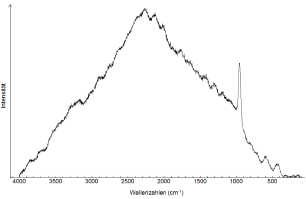Cu | |
| GB14 + Bi |  | |
| 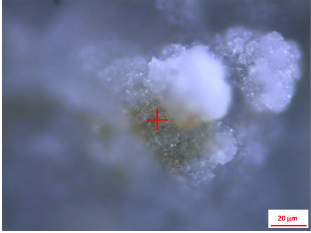 | 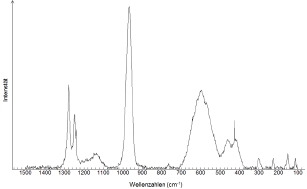Bismutphosphat unbek. Zusammensetzung | |

**Figure S9.** Raman spectroscopy of the GB14 + Bi and GB14 + Cu- coatings.

| Picture of the Measuring Point | Associated Raman-Spectrum | |  |
| --- | --- | --- | --- |
| HA + Cu |  | |  |
| 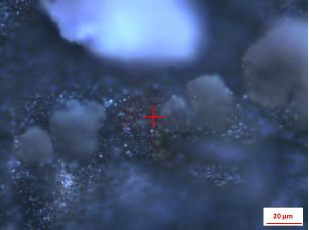 | 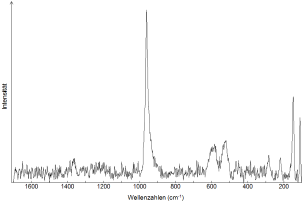HA mit CuO. Cu_2_O | |  |
| HA + Ag | |  | |
| 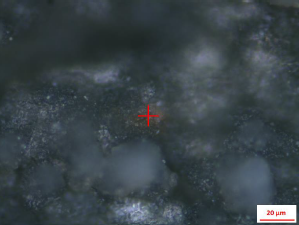 | | 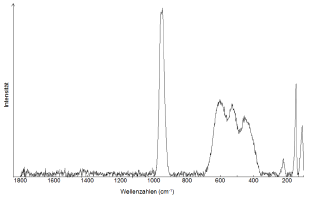Ag_3_PO_4_ und HA | |
| 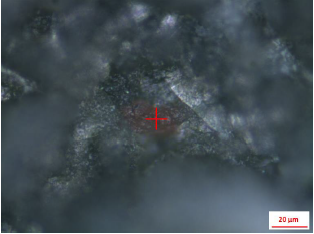 | | 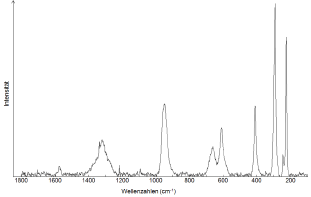 Fe_2_O_3_ und HA | |

**Figure S10.** Ramanspektroscopy of the HA + Cu, HA + Ag coating.

| Picture of the measuring point | Associated Raman-Spectrum |
| --- | --- |
| HA + Bi |  |
| 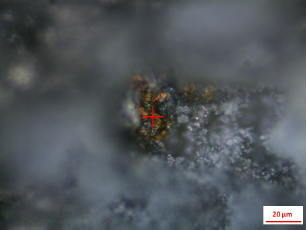 | 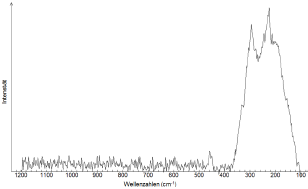α- Bi_2_O_3_ |
| 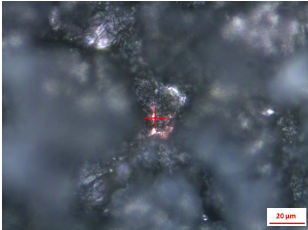 | 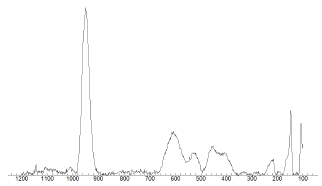HA mit α- Bi_2_O_3_ |

**Figure S11.** Ramanspectroscopy of the HA + Bi coating.

| Picture of the measuring point | | Associated Raman-Spectrum |
| --- | --- | --- |
| TCP + Cu |  | |
| 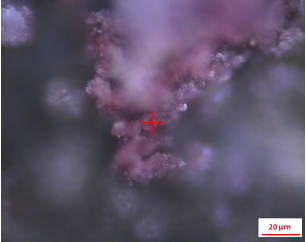 | 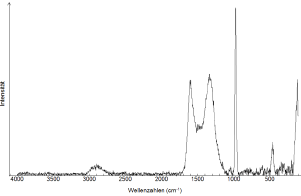amorpher Kohlenstoff. TCP bzw. Cu_3_(PO_4_)_2_ | |
| 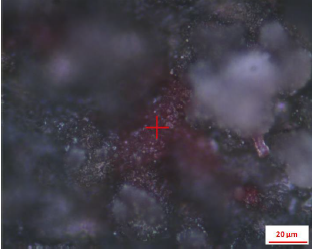 | 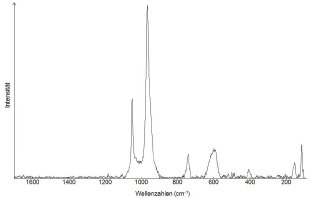TCP; Cu_3_(PO_4_)_2_. Cu(NO_3_)_2_ | |

**Figure S12.** Raman spectroscopy of the TCP + Cu coating.

5. SEM Images of the coatings

|  | **Cu** | **Ag** | **Bi** |
| --- | --- | --- | --- |
| **Bioglass** | 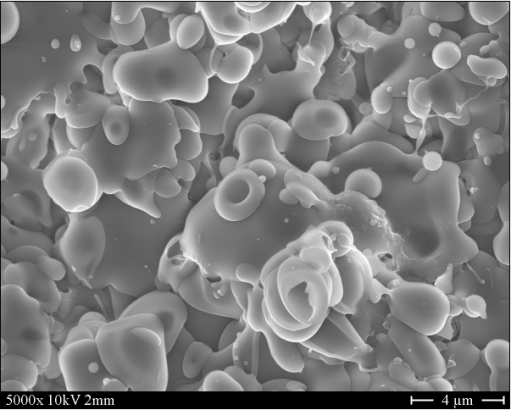  ****  **4 µm** | 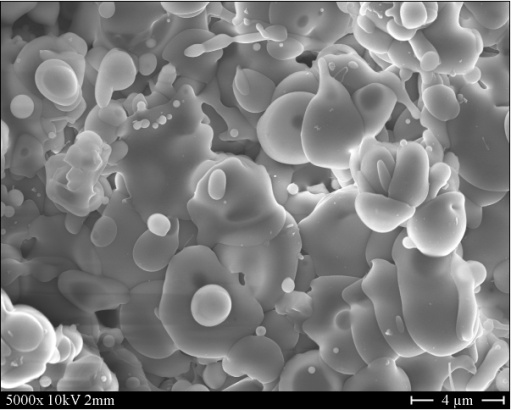  ****  **4 µm** | 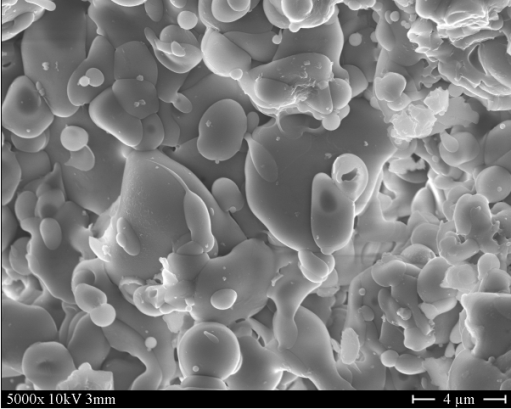  ****  **4 µm** |
| **TCP** | 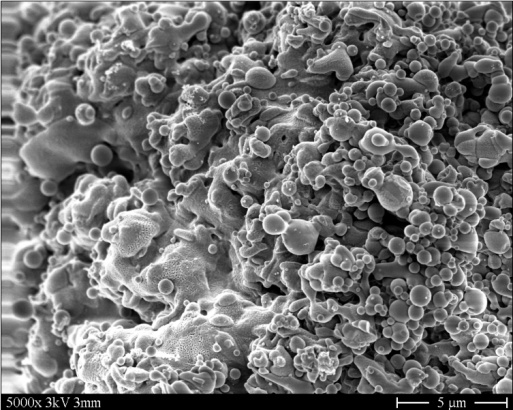  ****  **5 µm** | 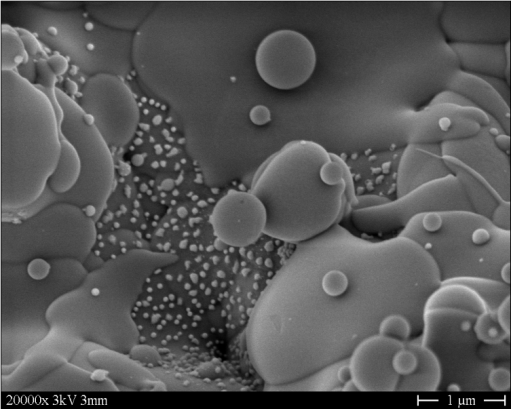  **1 µm**  **m**  ****  **1 µm** | 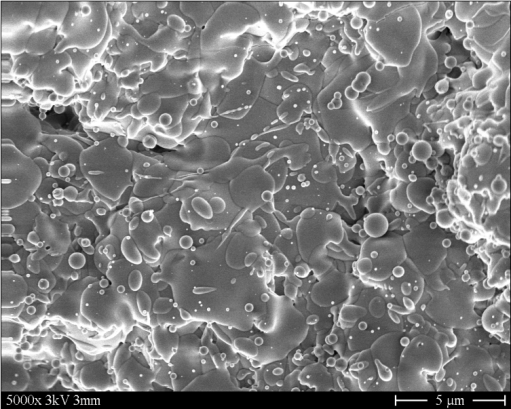  ****  **5 µm** |
| **HA** | 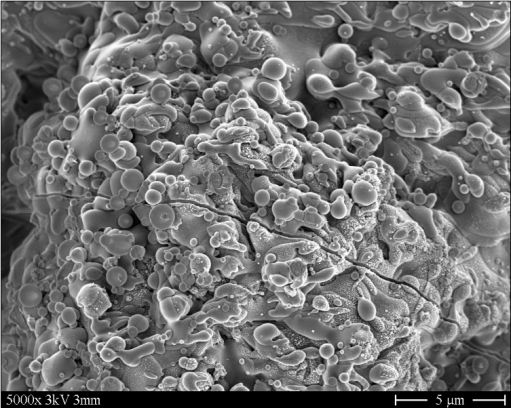  ****  **5 µm** | 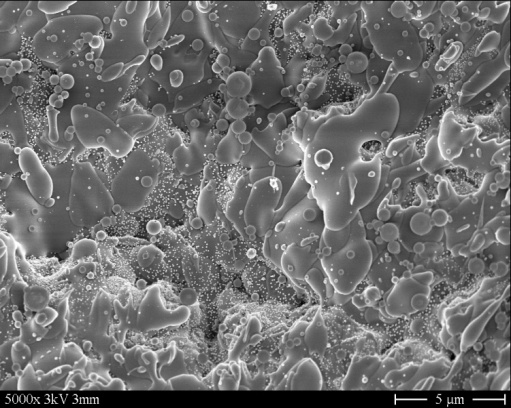  ****  **5 µm** | 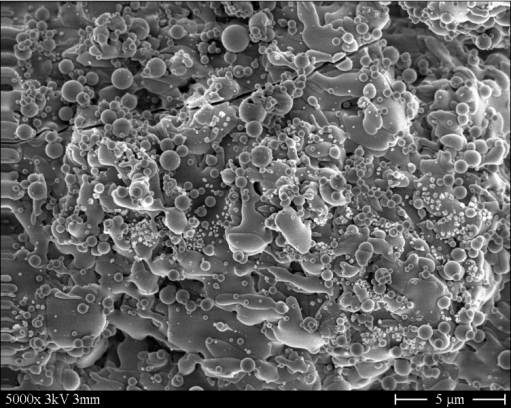  ****  **5 µm** |
| **GB14** | 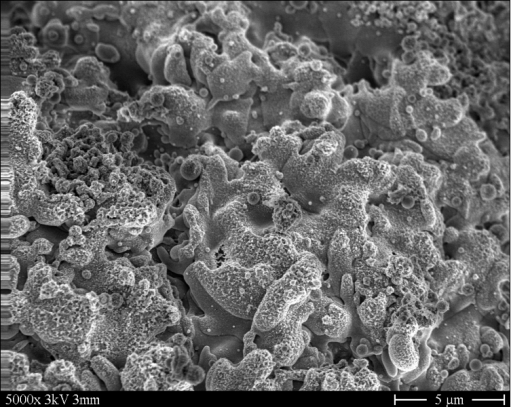  ****  **5 µm** | 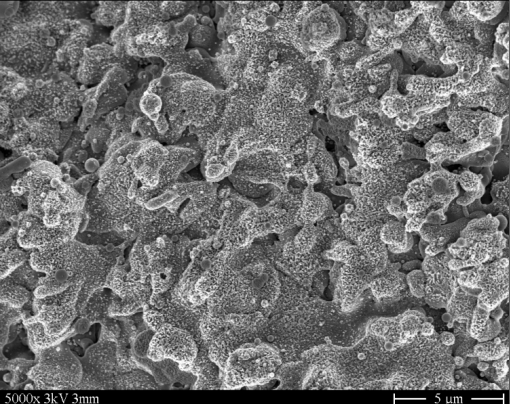  ****  **5 µm** |  |

**Figure S13.** SEM- Images of the coatings.

6. Release kinetics

**Table** S3. Metal ion release-kinetics values.

|  | **6h** | | | **24h** | | | **48h** | | | **72h** | | |
| --- | --- | --- | --- | --- | --- | --- | --- | --- | --- | --- | --- | --- |
| **Beschichtung** | **Release (ppb)** | **SD** | **µg/ml** | **Release (ppb)** | **SD** | **µg/ml** | **Release (ppb)** | **SD** | **µg/ml** | **Release (ppb)** | **SD** | **µg/ml** |
| **TCP + Cu** | 6.5 | 0.06 | 0.0065 | 4.8 | 0.31 | 0.0048 | <2 | 0.00 |  | <2 | 0.00 |  |
| **TCP + Ag** | <0.5 | 0.03 |  | <0.5 | 0.06 |  | <0.5 | 0.1 |  | <0.5 | 0.09 |  |
| **TCP + Bi** | 6.1 | 0.49 | 0.0061 | 1.9 | 0.64 | 0.0019 | 3.6 | 2.11 | 0.0036 | 5.9 | 0.18 | 0.0059 |
| **HA + Cu** | <2 | 0.00 |  | <2 | 0.00 |  | <2 | 0.00 |  | <2 | 0.00 |  |
| **HA + Ag** | <0.5 | 0.00 |  | <0.5 | 0.00 |  | <0.5 | 0.09 |  | <0.5 | 0.00 |  |
| **HA + Bi** | 4.6 | 0.28 | 0.0046 | 4.6 | 0.65 | 0.0046 | 4.3 | 1.69 | 0.0043 | 5.4 | 0.45 | 0.0054 |
| **GB14 + Cu** | 3.7 | 0.19 | 0.0037 | 5.4 | 0.15 | 0.0054 | 7.9 | 0.2 | 0.0079 | 14 | 0.35 | 0.0140 |
| **GB14 + Ag** | <0.05 | 0.00 |  | <0.5 | 0.00 |  | 1.2 | 0.08 | 0.0012 | 2.7 | 0.2 | 0.0027 |
| **GB14 + Bi** | 11.6 | 1.43 | 0.0116 | 7.2 | 1.04 | 0.0072 | 6.0 | 1.735 | 0.0060 | 7.1 | 0.71 | 0.0071 |
| **Bioglas + Cu** | 6.4 | 0.09 | 0.0064 | 6.4 | 0.15 | 0.0064 | 7.5 | 0.52 | 0.0075 | 11.4 | 1.58 | 0.0114 |
| **Bioglas + Ag** | <0.5 | 0.00 |  | <0.5 | 0.00 |  | <0.5 | 0.00 |  | <0.5 | 0.00 |  |
| **Bioglas + Bi** | 6 | 0.87 | 0.0060 | 4.8 | 1.47 | 0.0048 | 3.8 | 1.44 | 0.0038 | 4.1 | 1 | 0.0041 |

**Table S4.** Ceramic release in mmol/L. after 6. 24. 48 and 72 h.

|  | 6 h | | | | 24 h | | | | 48 h | | | | 72 h | | | |
| --- | --- | --- | --- | --- | --- | --- | --- | --- | --- | --- | --- | --- | --- | --- | --- | --- |
|  | **Ca^2+^** | **K^+^** | **Na^+^** | **PO_4_^3-^** | **Ca^2+^** | **K^+^** | **Na^+^** | **PO_4_^3-^** | **Ca^2+^** | **K^+^** | **Na^+^** | **PO_4_^3-^** | **Ca^2+^** | **K^+^** | **Na^+^** | **PO_4_^3-^** |
| TCP + Cu | <0.07 | <1.50 | <80 | <0.05 | <0.11 | <1.50 | <80 | 0.10 | <0.12 | <1.50 | <80 | 0.10 | <0.11 | <1.50 | <80 | 0.10 |
| TCP + Ag | <0.15 | <1.50 | <80 | 0.10 | <0.12 | <1.50 | <80 | <0.05 | <0.01 | <1.50 | <80 | <0.05 | <0.09 | <1.50 | <80 | <0.05 |
| TCP + Bi | <0.07 | <1.50 | <80 | 0.10 | <0.07 | <1.50 | <80 | 0.10 | <0.07 | <1.50 | <80 | 0.10 | <0.08 | <1.50 | <80 | 0.10 |
| HA + Cu | 0.22 | <1.50 | <80 | <0.05 | <0.19 | <1.50 | <80 | <0.05 | <0.14 | <1.50 | <80 | <0.05 | <0.11 | <1.50 | <80 | <0.05 |
| HA + Ag | <0.09 | <1.50 | <80 | 0.10 | <0.11 | <1.50 | <80 | 0.10 | <0.12 | <1.50 | <80 | 0.10 | <0.12 | <1.50 | <80 | 0.10 |
| HA + Bi | <0.11 | <1.50 | <80 | <0.05 | <0.12 | <1.50 | <80 | 0.10 | <0.14 | <1.50 | <80 | 0.10 | <0.02 | <1.50 | <80 | <0.05 |
| GB14 + Cu | <0.06 | <1.50 | <80 | 0.10 | <0.12 | <1.50 | <80 | 0.10 | <0.09 | <1.50 | <80 | 0.10 | <0.02 | <1.50 | <80 | 0.10 |
| GB14 + Ag | 0.23 | <1.50 | <80 | <0.05 | 0.22 | <1.50 | <80 | <0.05 | <0.19 | <1.50 | <80 | <0.05 | <0.18 | <1.50 | <80 | <0.05 |
| B14 + Bi | <0.08 | <1.50 | <80 | <0.05 | <0.10 | <1.50 | <80 | 0.10 | <0.13 | <1.50 | <80 | 0.10 | <0.13 | <1.50 | <80 | 0.10 |
| Bioglas + Cu | <0.10 | <1.50 | <80 | 0.10 | <0.11 | <1.50 | <80 | 0.10 | <0.12 | <1.50 | <80 | 0.10 | <0.12 | <1.50 | <80 | 0.10 |
| Bioglas + Ag | <0.07 | <1.50 | <80 | 0.10 | <0.07 | <1.50 | <80 | 0.10 | <0.09 | <1.50 | <80 | 0.10 | <0.09 | <1.50 | <80 | 0.10 |
| Bioglas + Bi | <0.19 | <1.50 | <80 | <0.05 | <0.18 | <1.50 | <80 | <0.05 | <0.19 | <1.50 | <80 | <0.050 | 0.23 | <1.50 | <80 | <0.05 |
| H_2_O | <0.00 | <1.50 | <80 | <0.05 | <-0.00 | <1.50 | <80 | <0.05 | <0.01 | <1.50 | <80 | <0.050 | <-0.01 | <1.50 | <80 | <0.05 |
| Titan glatt | <0.00 | <1.50 | <80 | <0.05 | <0.00 | <1.50 | <80 | <0.05 | <0.01 | <1.50 | <80 | <0.050 | <-0.01 | <1.50 | <80 | <0.05 |
| Titan rau | <0.01 | <1.50 | <80 | <0.05 | <0.00 | <1.50 | <80 | <0.05 | <0.00 | <1.50 | <80 | <0.050 | <-0.00 | <1.50 | <80 | <0.05 |


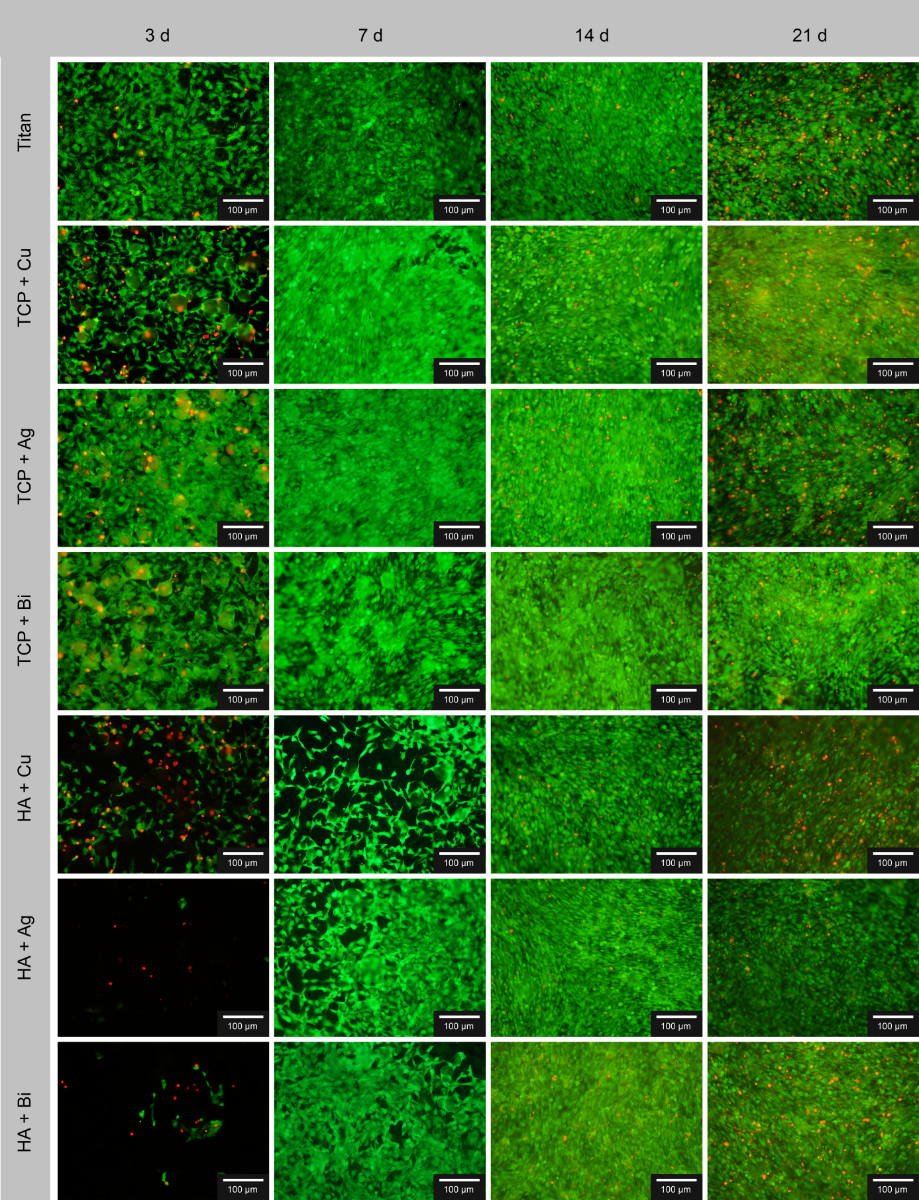


**Figure S14.** Dead/live assay. cell expansion on the metal doped hydroxylapatite. tricalciumphosphate. GB14 and bioglass coatings after 3. 7. 14. 21 days. 10× magnification. Living cells appear green. dead cells appear red.

| 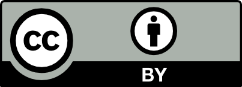 | © 2019 by the authors. Submitted for possible open access publication under the terms and conditions of the Creative Commons Attribution (CC BY) license (http://creativecommons.org/licenses/by/4.0/). |
| --- | --- |
